# Ferroptosis‐Based Peripheral Immune Dysregulation and Diagnostic Signatures in Parkinson's Disease: An RNA Transcriptomic and Single‐Cell Immune Sequencing Analysis

**DOI:** 10.1096/fj.202503867R

**Published:** 2026-08-02

**Authors:** Lei Cheng, Jing Zhang, Wen‐Ting Shi, Qing Ye, Chen Huang, Zhong‐Yan Zhou

**Affiliations:** ^1^ Longhua Hospital Shanghai University of Traditional Chinese Medicine Shanghai P.R. China; ^2^ Dr. Nesher's Biophysics Laboratory for Innovative Drug Discovery, State Key Laboratory of Quality Research in Chinese Medicine, Faculty of Chinese Medicine Macau University of Science and Technology Taipa China; ^3^ State Key Laboratory of Pharmaceutical Biotechnology, Department of Pharmacology and Pharmacy The University of Hong Kong Hong Kong SAR China; ^4^ Cardiac and Vascular Center The University of Hong Kong‐Shenzhen Hospital Shenzhen P.R. China

**Keywords:** diagnostic model, ferroptosis, immune infiltration, immune microenvironment, Parkinson's disease

## Abstract

Parkinson's disease (PD) is a common neurodegenerative disorder in which ferroptosis and immune dysregulation have been implicated. However, the crosstalk between ferroptosis‐related transcriptional signatures and peripheral immune alterations in PD remain unclear. Herein, we integrated bulk transcriptomic datasets and a peripheral blood single‐cell dataset to identify ferroptosis‐related genes associated with PD and to evaluate their diagnostic potential. Initially, we identified 16 abnormally expressed ferroptosis‐related genes (AEFRGs) associated with peripheral immune cell infiltration in PD. Then a LASSO‐derived 15‐gene signature was established, which showed high discriminatory performance in the discovery dataset and in two validation datasets, including an independent blood dataset and a substantia nigra dataset. Consensus clustering of PD samples based on 276 ferroptosis‐related genes (FRGs) stratified PD patients into three molecular subtypes with different diagnostic scores, immune scores, immune‐related factors, and cell‐death/oxidative‐stress pathway enrichment. The imumue cell infiltration analysis indicated low level of macrophage and high level of B cell in PD patients' perioheral blood. Peripheral blood single‐cell analysis localized ferroptosis‐related transcriptional features mainly related to NK cells, CD4^+^ T cells and CD8^+^ T cells in PD patients with significant difference of RPL8 and ATM, and identified 9 key characteristic ferroptosis genes (CFGs). In rotenone‐treated human neuroblastoma SH‐SY5Y cells, the CFGs inculding XBP1, SCP2, GABARAPL1, DUSP1, HSPA5 and HERPUD1 were increased, whereas UBC, RPL8 and ATM were not significantly changed. Collectively, these findings suggest that ferroptosis‐related transcriptional signatures may reflect peripheral immune dysregulation and provide candidate diagnostic markers for PD.

## Graphical abstract

This study explored the crosstalk between ferroptosis‐related genes and peripheral immune alterations in Parkinson's disease. We constructed a gene signature and classified molecular subtypes, and further pinpointed key ferroptosis‐associated molecules in immune cells and neuronal cells.

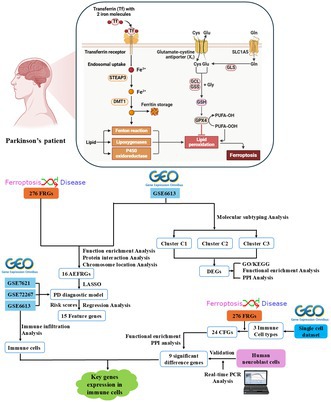

## 1 Introduction

Parkinson's disease (PD) is a central nervous system (CNS) disease featured by the degeneration and death of dopaminergic neurons in the substantia nigra region of the midbrain [1, 2]. PD is characterized by movement disorders including static tremor, bradykinesia and postural balance disorder, which might be accompanied by other non‐motor disorders, such as sleep disorder, memory decline and cognitive impairment [3]. At present, the global case of PD patients has exceeded 6 million, with a 2.5‐fold increase in the past 20 years, and the number is still increasing rapidly [4]. Mitochondrial dysfunction and abnormal activation of microglia play a crucial role in the casuses of PD. The immune and inflammatory reaction has received more and more attention during the selective death of dopaminergic neurons [5, 6]. However, there is still a lack of satisfactory treatment approaches for PD due to its unclear pathogenesis and poor early diagnosis.

Ferroptosis is a new form of programmed cell death that is different from apoptosis, necrosis, autophagy, and other forms in morphology, biochemistry and gene expression [7, 8]. Ferroptosis is characterized by iron dependence/overload and lipid oxidation and is prominently associated with the occurrence and development of tumors and degenerative diseases [9]. Jiang H et al. reported that the iron levels in the substantia nigra were significantly higher in PD patients than in normal subjects regardless of the remaining brain tissue, suggesting abnormal iron metabolism is closely related to the development of PD [10]. The iron accumulation in PD patients may cause degeneration of substantia nigra neurons by inducing ferroptosis, ultimately leading to the occurrence and progression of PD. Dexter DT et al. compared the content of lipid peroxides between the brain tissue of 18 PD patients and 19 normal subjects, and found that the level of lipid peroxides in the substantia nigra of PD patients was significantly higher than that of the control group, which provides direct evidence of ferroptosis in the brain of PD patients [11]. But the ferroptosis‐related pathways and related underlying mechanisms in PD still needs further research.

In this study, we extract the gene expression profiling and single‐cell sequencing datasets from the public databases to explore the ferroptosis‐related key genes and the role of ferroptosis in the development of PD, which have also been verified on an experimental PD model using human neuroblastoma cells.

## 2 Materials and Methods

### 2.1 Download and Preparation of Data

We used the “GEOquery” package of R [12] to download the PD expression profile datasets GSE6613 [13], GSE72267 [14] and GSE7621 [15] from the Gene Expression Omnibus (GEO) (https://www.ncbi.nlm.nih.gov/geo/). The samples in these dataset are all from *Homo sapiens* , with GSE6613 based on the platform GPL96, including 50 PD and 22 health samples, sourced from the peripheral blood; GSE72267 based on platform GPL571, including 40 PD and 19 health samples, sourced from blood; GSE7621 based on the platform GPL570, including 16 PD and 9 health samples, sourced from the substantia nigra. GSE6613 was selected as the discovery dataset because it contained a relatively larger whole‐blood cohort and was consistent with the aim of identifying accessible diagnostic biomarkers. GSE7621, which was derived from substantia nigra tissue and had a smaller sample size, was used as an independent validation dataset rather than a discovery dataset. This design allowed us to test whether the blood‐derived signature retained discriminatory relevance in a PD‐affected brain region, while avoiding overinterpretation of blood markers as direct surrogates of CNS pathology. We also downloaded the 10× Genomics data of single‐cell sequencing data (scRNA) referring to a previous study [16] which consists of 8 PD and 6 health samples for subsequent analysis, all sourced from blood (Table 1).

**TABLE 1:** The 4 datasets were emplyed for data analysis.

No.	Dataset	Platform	Samples	Source	Link
1	GSE6613	GPL96	50 PD patients and 22 controls	Blood	https://www.ncbi.nlm.nih.gov/geo/query/acc.cgi?acc=GSE6613
2	GSE72267	GPL571	40 PD patients and 19 controls	Blood	https://www.ncbi.nlm.nih.gov/geo/query/acc.cgi?acc=GSE72267
3	GSE7621	GPL570	16 PD patients and 9 controls	Substantia nigra	https://www.ncbi.nlm.nih.gov/geo/query/acc.cgi?acc=GSE7621
4	scRNA	10 × Genomics	8 PD patients and 6 controls	Blood	https://zenodo.org/record/3993994

We performed data processing and annotation on the GSE6613, GSE72267 and GSE7621 dataset by using the “hgu133a.db”, “hgu133a2.db” and “hgu133plus2.db” packages [17], respectively. The HUGO Gene Nomenclature Committee (HGNC) [18] provided a unique, standard and widely disseminated symbol for each human genome, and its mRNA gene annotation files were used to build the mRNA expression profile. FerrDb database stores the markers and regulatory factors of all ferroptosis [19], from which we obtained 276 ferroptosis‐related genes (FRGs) for subsequent analysis.

### 2.2 Functional Analysis of Ferroptosis‐Related Genes

We obtained the expression matrix of FRGs in the dataset GSE6613 and used “wilcox.test” [13] in R to obtain genes with significant expression difference (*p* < 0.05), which are named abnormally expressed FRGs (AEFRGs). We visualized the expression difference of AEFRGs between PD patients and normal samples with a box graph. The position of AEFRGs on chromosomes was mapped with the R package “RCircos” [20]. Metascape provided data from over 40 gene function annotation databases and diverse visualization methods [21], aiming to analyze the function enrichment of the AEFRGs with the criteria of *p* < 0.01, a minimum count of 3, and an enrichment factor > 1.5. The “corrplot” package was introduced to perform correlation analysis and visualize by a correlation heat map. The “GOSemSim” package [22] was used to conduct functional similarity analysis to explore the interaction between the AEFRGs. We uploaded the obtained AEFRGs into “Networkanalysis” [23] to obtain the mRNA‐TF (Translation Factor) interaction network based on JASPAR [24]. Subsequently, based on TarBase V8.0 [25], the mRNA‐miRNA interaction network was obtained. We obtained the RNA‐binding protein (RBP)‐mRNA interaction network from ENCORI, which is the version 3.0 of starBase and stores small molecules interacting with mRNAs [26].

### 2.3 Evaluation of Immune Cell Infiltration and Its Correlation With Differentially Expressed Ferroptosis Genes

We adopted the tumor microenvironment analysis method for exploration of the immue features in PD peripheral blood. Briefly, CIBERSORT [27] was used to deconvolute the expression matrix and estimate the composition and abundance of immune cells. We downloaded immune cell files from the official website of CIBERSORT and obtained the immune cell infiltration matrix based on the GSE6613 dataset. Because GSE6613 was a whole‐blood dataset and the CIBERSORT reference matrix mainly represented peripheral immune cell signatures, the deconvolution results were interpreted as estimated peripheral immune cell composition rather than CNS immune infiltration. Brain‐resident immune populations, such as microglia, were not evaluated by this analysis. We visualized the composition of immune cells in the PD and health groups with box plots by using the R “ggplot2” package. We also calculated and visualized the correlation between 21 types of immune cells, which except mast cell actived from the standard 22 types of immune cells, and between AEFRGs and the immune cell infiltration by heat maps.

### 2.4 Molecular Subtype Analysis Based on Ferroptosis Related Genes

The R “ConsensusClusterPlus” package was used to conduct the algorithm of the PD samples in GSE6613 dataset and select the best clustering subtypes using consistency Clustering [28]. The differentially expressed genes (DEGs) of clusters were screened using the “Limma” package [29] with the criteria of *p* value < 0.05 and |log2FC| > 1. Furthermore, the R “estimate” package [30] was used to calculate the ESTIMATE score, immune score, matrix score and tumor purity on the GSE6613 dataset and visualized by using ggplot2 package to draw violin diagrams and box plots. Finally, we obtained immunosuppressive factors, immunostimulatory factors, chemokines and their receptors from the TISIDB database [31] and the differences among substypes were presented by box plots.

### 2.5 Functional Enrichment Analysis of Subtypes

The annotation of gene function and related pathways were based on Gene Ontology (GO) [32] and Kyoto Encyclopedia of Genes and Genomes (KEGG) enrichment analysis [33]. We used Metascape to perform GO/KEGG enrichment of the DEGs between subtypes of GSE6613 and screened the items with *p* < 0.05. Single sample gene set enrichment analysis (ssGSEA) [34] was used to calculate the enrichment score of each sample, which represents the degree that a specific gene set are coordinated up or down regulation. We used “GSVA” package to perform ssGSEA on the GSE6613 dataset and selected reference gene set from the Molecular Signatures Database (MSigDB) [35]. A box plot was drawn to visualize pathways with significant difference between subtypes.

### 2.6 Construction and Verification of PD Diagnostic Model

The Least Absolute Shrinkage and Selection Operator (LASSO) [36], which is a shrinkage estimation method, was used to construct a penalty function to compress the coefficients of variables and make certain regression coefficients zero, thereby achieving the goal of variable selection. We used “glm” function in R to conduct feature selection, screen PD diagnostic markers, and build a classifier model. According to the model, each sample in GSE6613, GSE72267 and GSE7621 is scored for risk. We used the R package “pROC” [37] to draw the Receiver operating characteristic (ROC) of the model and calculate the Area Under Curve (AUC), which aims to assess the accuracy of the risk score for distinguish the PD samples in GSE6613, GSE72267 and GSE7621 datasets. Furthermore, the distribution of risk scores in the PD and health groups was demonstrated using violin charts in GSE6613, GSE72267 and GSE7621 datasets.

### 2.7 Single‐Cell RNA‐Sequencing Data Processing, Clustering and Annotation PD

The Single‐Cell RNA‐Sequencing (scRNA) data analysis is based on the Seurat [38] which is an integrated software package developed by New York Genome Center. We downloaded the single‐cell data and used this package to select cells with more than 200 genes (each gene was included by more than 10 cells). Linear dimensionality reduction was performed by the Principal Component Analysis (PCA) method, and the optimal significance dimension was selected by the “ElbowPlot” and “JackStrawPlot” functions. We explored and visualized the clustering of cells using the Uniform Manifold Approximation and Projection (UMAP) algorithm based on the optimal dimension. The “SingleR” package [39], which is based on the “DatabaseImmuneCellExpressionData” in “celldex” [40], was used as a reference to annotate the cell clusters and visualized the clusters in the PD and health groups.

### 2.8 Differential Expression Analysis and Functional Annotation of Cell Subtypes in scRNA Data

We used the “FindMarkers” function in Seurat to analyze the differential expression genes of ferroptosis with the screening criteria: (a) Ferroptosis gene was expressed in at least 10% of the genes in the two groups; (b) |log2FC| > 0.25; (c) FDR < 0.05. The “DoHeatmap” function was used to draw a thermogram to show the expression pattern of ferroptosis genes in different cell types. The difference of FRGs between different cell types was presented as a dot‐plot by the “DotPlot” function. The “FeaturePlot” function was further used to visualize the expression of FRGs. A violin diagram was plotted to verify the difference of FRGs obtained from GSE6613 between PD and healthy groups in scRNA data, and “wilcox.test” was applied to test the significance. “ClusterProfiler” package in R [41], which supports both GO and KEGG enrichment analysis with excellent visualization capabilities, was emplyed in this study with screening criteria of *p* < 0.05. STRING [42] database, which stores interactions between known and predicted proteins, was used to construct a protein–protein interaction (PPI) network for the FRGs with minimum required interaction score: low confidence (0.150) as the standard.

### 2.9 Real‐Time PCR Validation

The human neuroblastoma cells SH‐SY5Y (ATCC, CRL‐2266) was cultured in Dulbecco's modified Eagle's medium (DMEM) supplemented with 10% fetal bovine serum (FBS) and 1% penicilin‐streptomycin (PS) in incubator (37°C, 5% CO_2_). The SH‐SY5Y cells were treated with or without 50 μM rotenone for 24 h and followed by total RNA extraction using Tripure kit (Roche, Manneheim, Germany). The total RNA was transcribed into single‐strand cDNA using Transcriptor First Strand cDNA Synthesis Kit (Roche, Manneheim, Germany). SYBGREEN PCR Master Mix (Roche, Manneheim, Germany) was used for real‐time detection of the PCR products that were generated by specific gene primers (Table 2) based on the Light Cycle 96 platform (LC96, Roche, Germany). The relative mRNA expression of 9 key CFGs were normalized by the internal control GAPDH and calculated by 2^‐^^ΔΔCt^ method.

**TABLE 2:** The specific primers were used for related genes expression analysis in Real‐time PCR.

Gene ID	Gene	Forward primer	Reverse primer
NM_005347.5	HSPA5	5′‐CACTCCTGAAGGGGAACGTC‐3′	5′‐TCAAAGACCGTGTTCTCGGG‐3′
NM_031412.4	GABARAPL1	5′‐AGGGTCCCCGTGATTGTAGA‐3′	5′‐TAAGGCGTCCTCAGGTCTCA‐3′
NM_002979.5	SCP2	5′‐TGTCCCACTTCAGATGGTGC‐3′	5′‐CCACAGCTTTGGATTGCAGG‐3′
NM_001393999.1	XBP1	5′‐GCTCAGACTGCCAGAGATCG‐3′	5′‐AGGCCATGAGTTTTCTCTCGT‐3′
NM_033301.3	RPL8	5′‐GCCACCGTTATCTCCCACAA‐3′	5′‐GGGTTTGTCAATTCGGCCA‐3′
XM_054368880.1	ATM	5′‐CTTGACCTGTGGTGAGCACT‐3′	5′‐TTGACGGCAGCAGATAAGCA‐3′
NM_001272103.2	HERPUD1	5′‐TGAATGCACAAGGCATCACG‐3′	5′‐AACGTCAGGAGGAGGACCAT‐3′
NM_004417.4	DUSP1	5′‐ACCACCACCGTGTTCAACTT‐3′	5′‐AGAGGTCGTAATGGGGCTCT‐3′
NM_021009.7	UBC	5′‐CAGCCGGGATTTGGGTCG‐3′	5′‐CACGAAGATCTGCATTGTCAAGT‐3′
NM_001289746.2	GAPDH	5′‐CACCATCTTCCAGGAGCGAG‐3′	5′‐GACTCCACGACGTACTCAGC‐3′

### 2.10 Statistical Analysis

All the analyses were based on R software (version 4.2.1). Wilcoxon Rank Sum Test was performed between two groups using “ggpubr” package [43], and Kruskal Wallis test was used for comparison among three groups. The Pearson correlation analysis was to calculate the correlation coefficients between two sets of parameters. *p* value < 0.05 was defined as significant difference.

## 3 Results

### 3.1 Data Profiling and Ferroptosis‐Related Genes Extraction

To verify ferroptosis involved in PD, we downloaded the gene expression matrices of GSE6613, GSE72267 and GSE7621 (Table 1) from the GEO official website and standardized the data. The gene annotation file is downloaded from HGNC, and after matching, a total of 11 952 mRNA was obtained from GSE6613 and GSE72267 datasets, and 16 898 mRNA obtained from GSE7621 dataset. Ferroptosis‐related genes (FRGs) are obtained from the FerrDb database and 276 FRGs are extracted (Supporting Information Dataset 1). We successfully standardized transcriptomic data from multiple platforms and obtained 276 FRGs, which contributes to the following identifying abnormally expressed FRGs (AEFRGs) and exploring their roles in PD.

### 3.2 Expression Characteristics of Abnormal Expressed Ferroptosis‐Related Genes in PD

**FIGURE 1:**
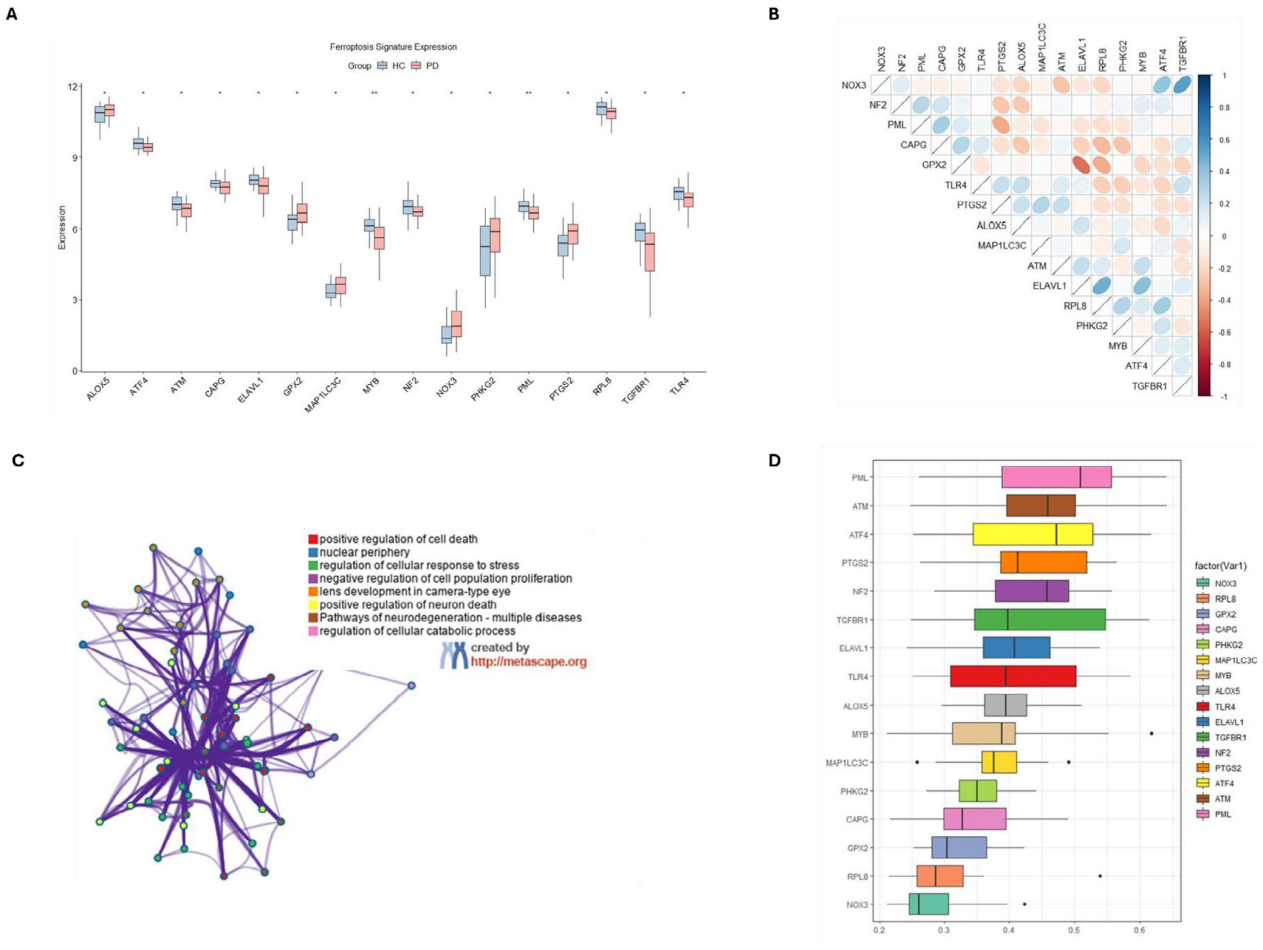
AEFRGs based on the data set GSE6613. (A) Box plot indicates the gene expression of the 16 AEFRGs; (B) The correlation heat map of these genes. Blue indicates positive correlation and red indicates negative correlation. The darker color indicated the stronger correlation; (C) The network diagram of the top 8 enrichment functions; (D) The results of function similarity analysis of 16 AEFRGs. AEFRGs, abnormally expressed ferroptosis‐related genes.

We profiled the expression matrix of 276 FRGs in the dataset of GSE6613 (Figure 1A), which showed that 16 FRGs were abnormally expressed (AEFRGs), with *ALOX5, GPX2, MAP1LC3C, NOX3, PHKG2 and PTGS2* upregulated while *ATF4, ATM, CAPG, ELAVL1, MYB, NF2, PML, RPL8, TGFBR1 and TLR4* downregulated in PD samples (Wilcoxon Rank Sum Test, *p* < 0.05) (Table 3). The thermogram of ferroptosis gene correlation showed that all the significantly correlated pairs, with *PTGS2* and *PML* negatively, *ELAVL1* and *GPX2* negatively, *RPL8* and *GPX2* negatively, *RPL8* and *ELAVL1* positively, and *TGFBR1* and *NOX3* positively (Figure 1B). We performed functional enrichment analysis on the AEFRGs and found that they were mainly enriched in “positive regulation of cell death”, “unclear localization”, “regulation of cellular response to stress”, “negative regulation of cell population promotion”, “positive regulation of neuron death”, “pathways of neuron generation‐multiple releases” and “regulation of cellular catalytic process” (Figure 1C). The function similarity analysis showed that *PML* was the most important gene (Figure 1D). To understand the regulatory characteristics of the FRGs from multiple levels, the chromosomal distribution and potential transcriptional regulatory networks were also analyzed. They described the distribution characteristics of the differentially expressed genes in the genome and the interaction networks of transcription factors and miRNAs, respectively, which provided an overall picture of how these FRGs might be regulated at the transcriptional and post‐transcriptional levels. The AEFRGs‐miRNA interaction network showed these 16 mRNA interacting with 318 miRNAs, the AEFRGs‐TF interaction network showed these 16 mRNA with 58 TFs, and the AEFRGs‐RBP interaction network showed these 16 mRNA interacting with 198 RBPs (Figure S1 and Supporting Information Dataset 2). The interaction networks of AEFRGs with miRNAs, TFs and RBPs further indicated complex regulatory mechanisms underlying the ferroptosis feature in PD. In summary, a total of 16 AEFRGs were identified, with 6 upregulated genes (*ALOX5, GPX2, MAP1LC3C, NOX3, PHKG2* and *PTGS2*) and 10 downregulated genes (*ATF4, ATM, CAPG, ELAVL1, MYB, NF2, PML, RPL8, TGFBR1* and *TLR4*) in PD samples. These genes are distributed across multiple chromosomes and enriched in cell death regulation, stress response and neurodegeneration‐related pathways, suggesting their potential involvement in PD pathogenesis through modulation of ferroptosis.

**TABLE 3:** The list of 16 abnormally expressed ferroptosis‐related genes.

Symbol	Name	Up‐or down‐regulated in PD patients
ALOX5	Arachidonate 5‐Lipoxygenase	↑
GPX2	Glutathione Peroxidase 2	↑
MAP1LC3C	Microtubule Associated Protein 1 Light Chain 3 Gamma	↑
NOX3	NADPH Oxidase 3	↑
PHKG2	Phosphorylase Kinase Catalytic Subunit Gamma 2	↑
PTGS2	Prostaglandin‐Endoperoxide Synthase 2	↑
ATF4	Activating Transcription Factor 4	↓
ATM	ATM Serine/Threonine Kinase	↓
CAPG	Capping Actin Protein, Gelsolin Like	↓
ELAVL1	ELAV Like RNA Binding Protein 1	↓
MYB	MYB Proto‐Oncogene, Transcription Factor	↓
NF2	NF2, Moesin‐Ezrin‐Radixin Like (MERLIN) Tumor Suppressor	↓
PML	PML Nuclear Body Scaffold	↓
RPL8	Ribosomal Protein L8	↓
TGFBR1	Transforming Growth Factor Beta Receptor 1	↓
TLR4	Toll Like Receptor 4	↓

*Note:* ↑, upregulation; ↓, downregulation.

**FIGURE 2:**
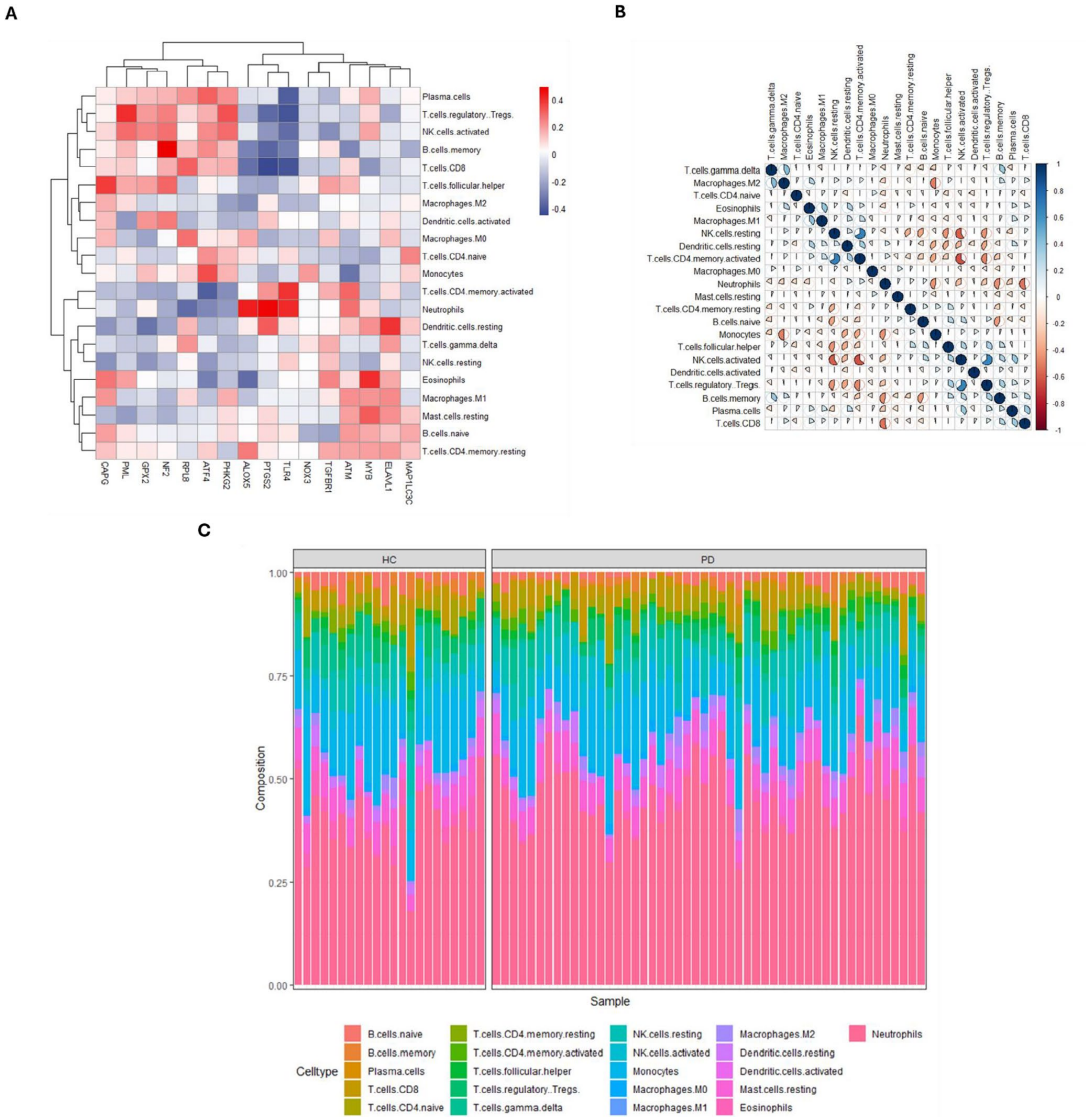
Immune cell infiltration based on dataset GSE6613 and correlation analysis. (A) Correlation heatmap of 21 types of immune cell infiltration and AEFRGs; Red‐positive, blue‐negative; (B) Correlation heatmap of 21 types of immune cell infiltration; Blue‐positive, red‐negative. The darker color indicated the stronger correlation; (C) Box plot of the proportion of immune cell infiltration.

### 3.3 Evaluation of Immune Cell Infiltration and Its Correlation With Abnormal Expressed Ferroptosis‐Related Genes

To explore the correlation between AEFRGs and 21 types of immune cells, our analysis showed all the correlated pairs. *plasma cells* with *TLR4* negatively and *ATF4* positively; *Tregs* with *TLR4* negatively, and *PHKG2* and *PML* positively; *B cells memory* with *NF2* positively; *Monocytes* with *ATF4* positively; *T cells CD4 memory actived* with *ATM* negatively and *TLR4* negatively; *Neutrophils* with *TLR4*, *PTGS2* and *ALOX5* positively; *Dendritic cells resting* with *PTGS2* and *ELAVL1* positively (Figure 2A). The results of immune cell related heat maps indicated certain correlations between these cells (Figure 2B). Immune infiltration level of *macrophages* was lower in the PD group, whereas *B cells* were relatively higher (Figure 2C). The CIBERSORT analysis suggested altered composition of estimated peripheral immune cell in PD blood samples. Specifically, *macrophage*‐like signatures were relatively lower, whereas *B‐cell* signatures were relatively higher in PD samples. These findings indicate peripheral immune remodeling associated with PD, but do not provide direct evidence of immune cell infiltration into the CNS.

### 3.4 PD Diagnostic Model Construction Based on Ferroptosis‐Related Genes and Verification

**FIGURE 3:**
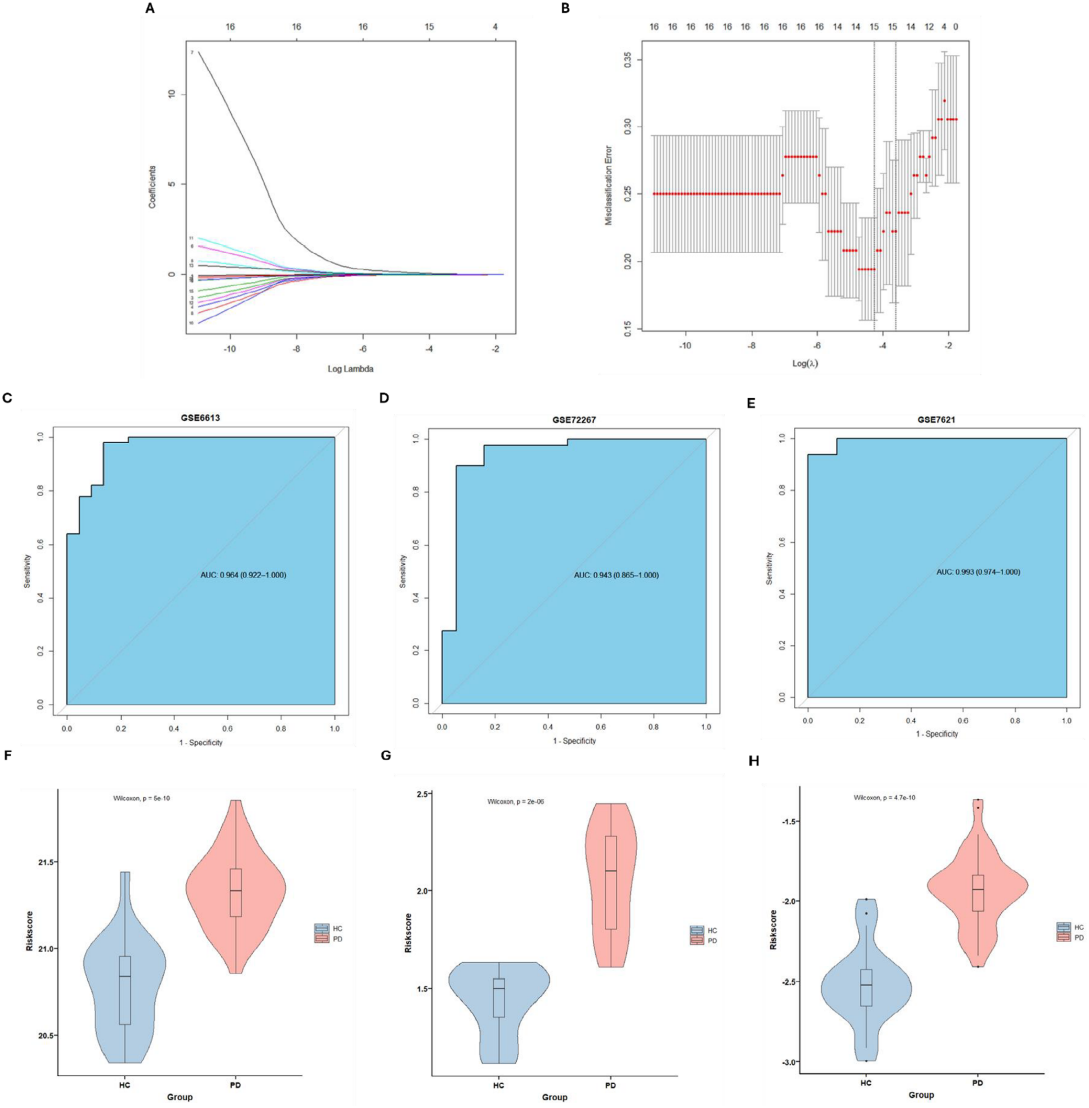
The diagnostic model of PD and validation. (A, B) In the dataset GSE6613, the LASSO logistic regression algorithm is used to screen PD diagnostic biomarkers; (C) ROC analysis of risk score in GSE6613, (D) GSE72267, and (E) GSE7621; (F) Violin diagram shows the risk score of GSE6613, (G) GSE72267, and (H) GSE7621. ROC: Receiver Operating Characteristic.

For the 16 AEFRGs in the dataset GSE6613, we used the LASSO logistic regression algorithm to identify the diagnostic markers and obtained a total of 15 genes (except *NOX3*) (Figure 3A,B). We constructed a PD diagnostic model based on multivariate logistic regression analysis using these 15 diagnostic markers. The datasets GSE72267 and GSE7621 were used as external validation cohorts. According to the above model, each sample in GSE6613, GSE72267 and GSE7621 was scored for diagnostic risk. The ROC results showed that the AUC of the risk score was 0.964 for GSE6613, 0.943 for GSE72267, and 0.993 for GSE7621 (Figure 3C–E). All the AUC values exceeded 0.9, indicating that the risk score had good diagnostic ability for PD and normal samples. The violin chart shows that the diagnostic risk scores of PD samples were significantly higher than those of the normal group samples in GSE6613, GSE72267 and GSE7621 datasets (Figure 3F–H) (Wilcoxon Rank Sum Test, *p* < 0.05). The risk score showed promising discriminatory performance in these datasets. However, considering the modest sample size and the relatively large number of genes in this model, the diagnostic signature should be regarded as exploratory and requires further prospective validation before clinical application.

### 3.5 Molecular Typing Analysis Based on Ferroptosis‐Related Genes

**FIGURE 4:**
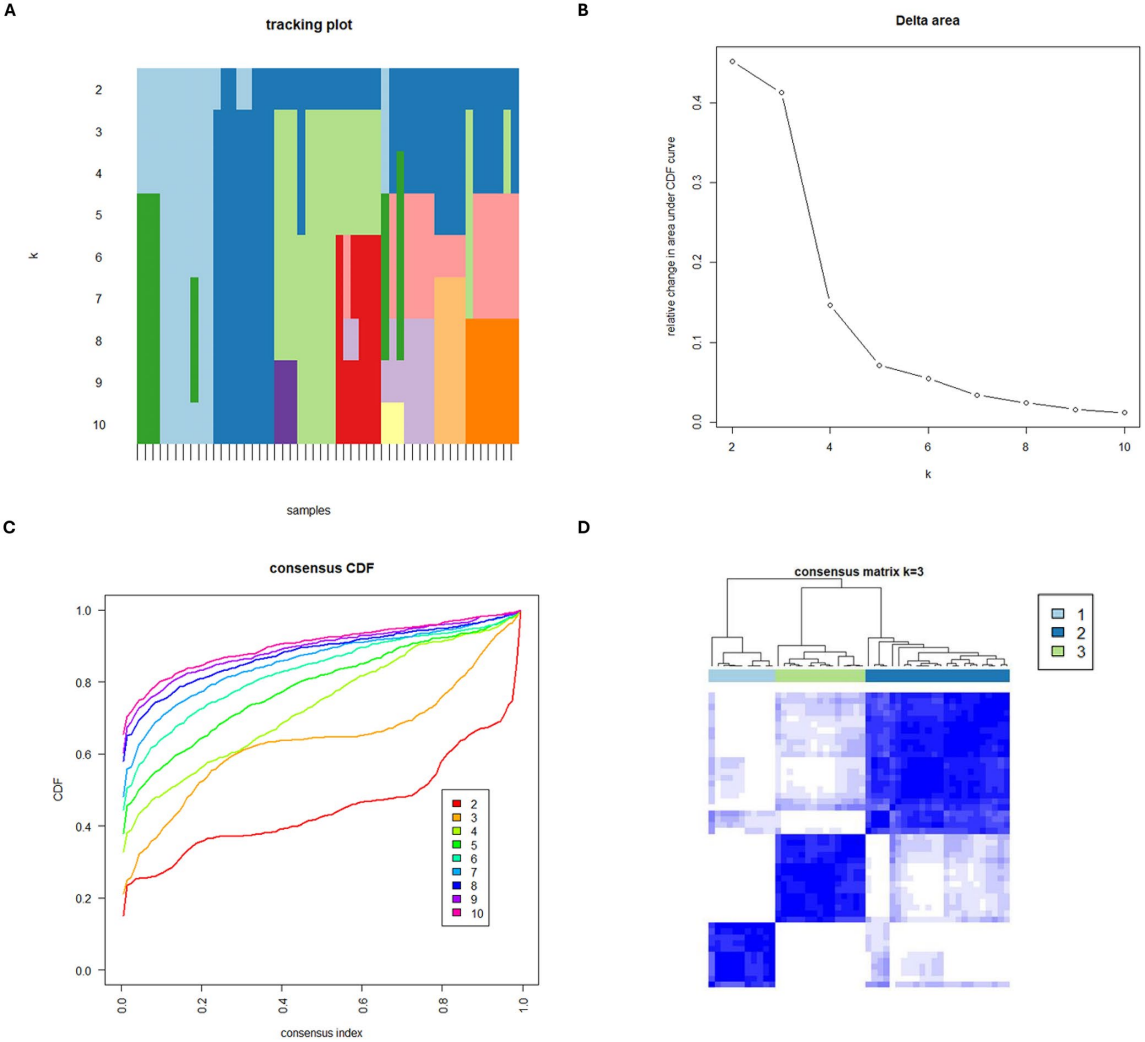
The consistency clustering analysis. (A) Tracking plot to display the clusters of the sample at each K; (B) The Cumulative distribution function (CDF); (C) The relative change in area under the CDF curve from 2 to 10 of k; (D) Consistency clustering diagram at *k* = 3.

**FIGURE 5:**
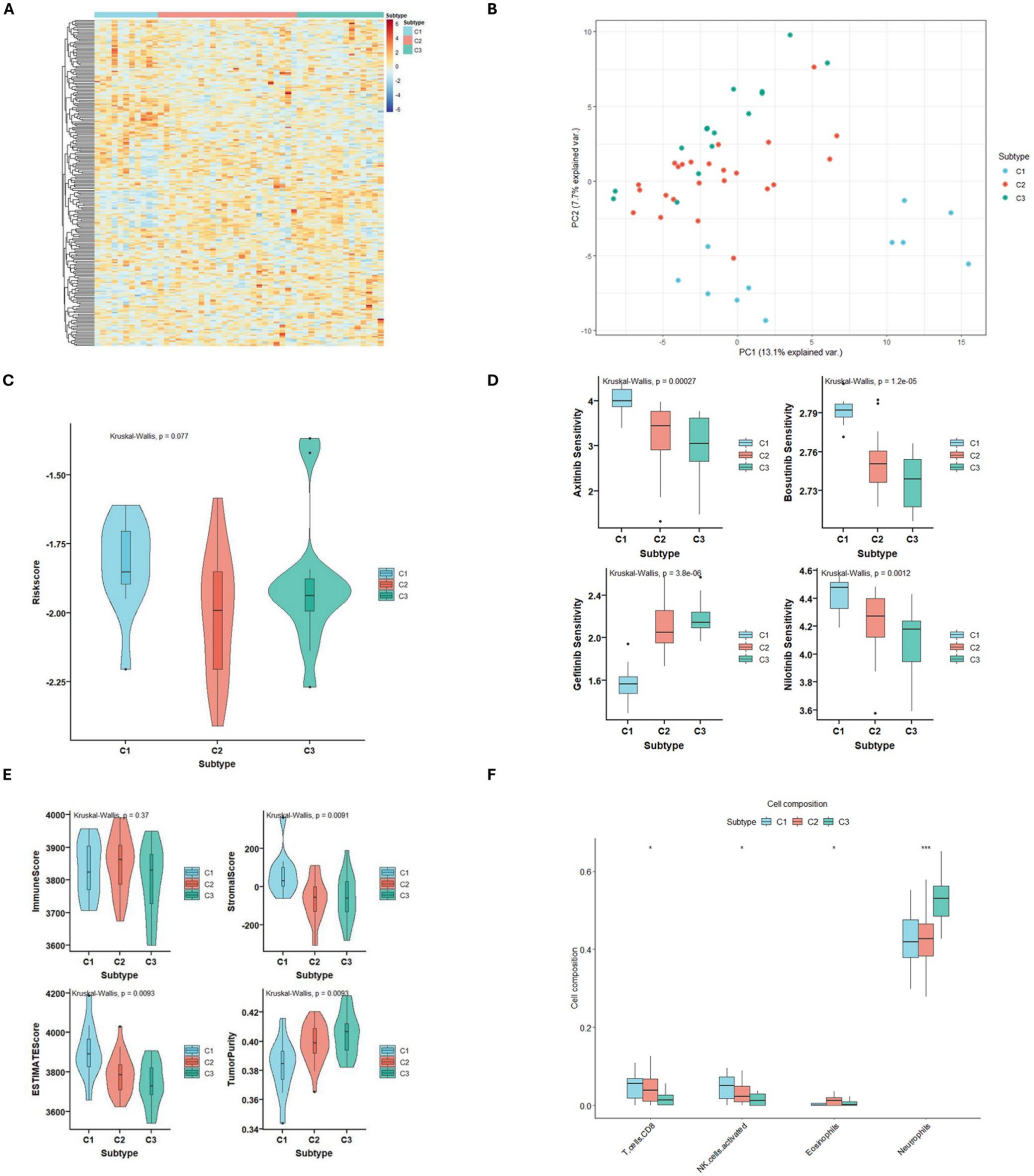
The PD molecular typing analysis and the immune features. (A) Heat map of FRGs; (B) PCA analysis based on FRGs; (C) Violin chart of risk score of 3 subtypes; (D) Box plot of drug sensitivity for Axitinib, Bosutinib, Gefitinib and Nilotinib among 3 subtypes; (E) Violin charts indicate the immune score, stromal score, STIMATE score and tumor purity of these 3 subtypes; (F) Box plot indicates the proportion of immune cell infiltration in these 3 subtypes.

**FIGURE 6:**
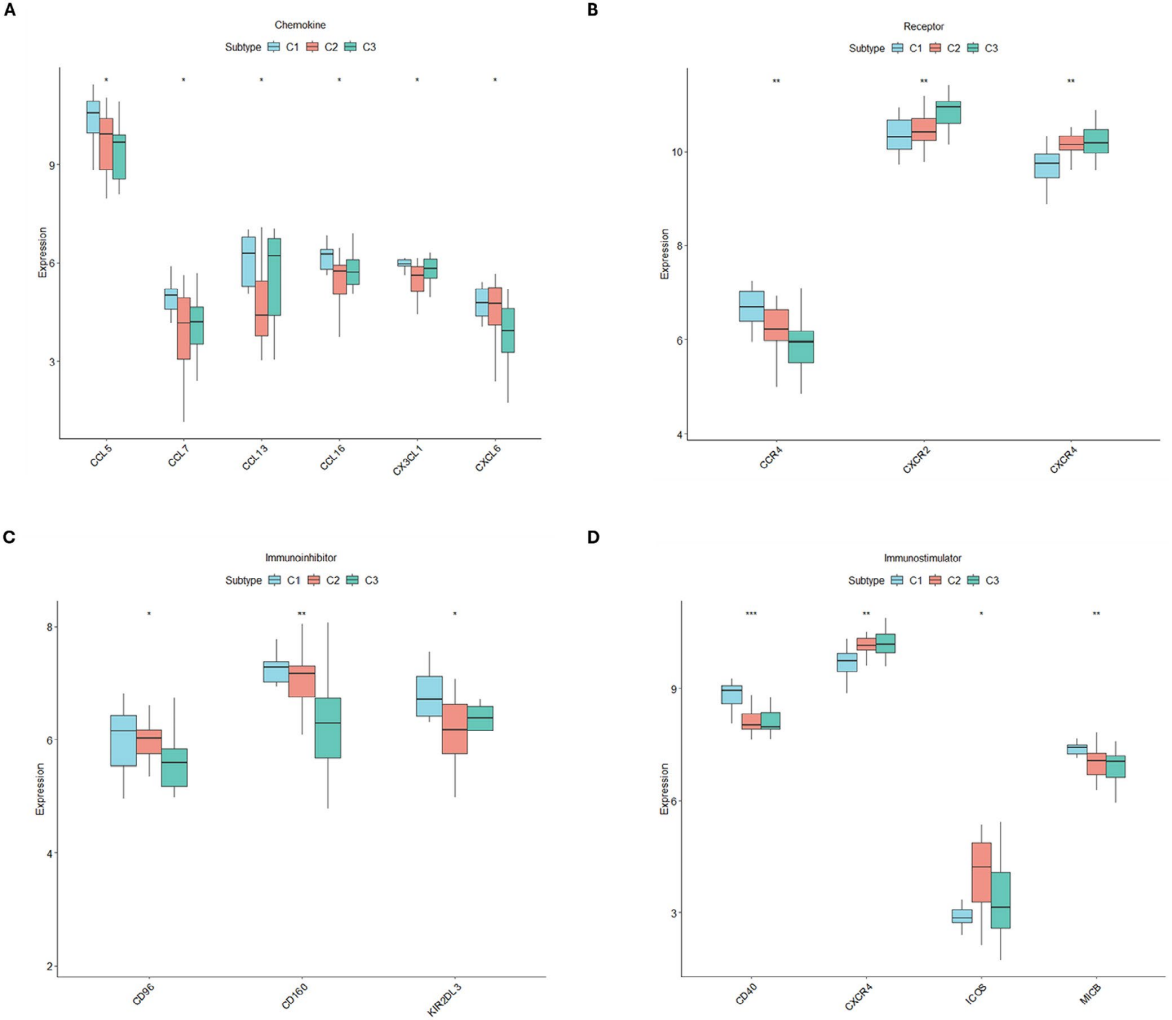
The differences of immune factors among 3 subtypes. (A) Box plot of chemotactic factors, (B) receptor between subtypes, (C) immunosuppressive factor and (D) immune stimulating factor.

We conducted consensus clustering of 50 PD samples in GSE6613 dataset based on 276 FRGs. A tracking plot displayed the clusters of the sample under each cluster number K (Figure 4A). According to the Delta area curve (DAC) (Figure 4B) and the cumulative distribution function (CDF) (Figure 4C), when the number of selected clusters is 3, the result is relatively stable and the clustering effect is the best (Figure 4D). We named these 3 clusters as subtypes C1, C2 and C3. According to the heat map of FRGs and PCA analysis, the expression of FRGs in these three subtypes was significantly different (Figure 5A,B). The violin plot of the PD diagnostic model showed the lowest score in the C2 group and the highest in the C1 group (Kruskal Wallis test, *p* = 0.077) (Figure 5C). C2 had the highest immune score. The ESTIMATE score and matrix score decreased sequentially while the purity score increased in the C1–C3 group (Figure 5E), demonstrating different immune scoring states in these three groups (Kruskal Wallis test, *p* < 0.05). Furthermore, the “pRRophetic” algorithm was used to predict the drug sensitivity of Axitinib, Bosutinib, Gefitinib and Nilotinib among subtypes. The box plot showed significant differences in drug sensitivity among the four drugs in the C1, C2 and C3 groups (Kruskal Wallis test, *p* < 0.05) (Figure 5D). Immune cell infiltration (Figure 5F) analysis revealed significant differences in the expression of *T cells CD8*, *NK cells activated*, *Eosinophels* and *Neutrophils* among 3 subtypes (Kruskal Wallis test, *p* < 0.05). The chemokine box plot addressed significant differences in expression between subtypes of *CCL5, CCL7, CCL13, CCL16, CX3CL1* and *CXCL6* (Figure 6A). The receptor box plot also showed significant differences in the expression of *CCR4, CXCR2* and *CXCR4* among 3 subtypes (Figure 6B). The immunosuppressive factor box plot indicated significant differences in the expression of *CD96, CD160* and *KIR2DL3* among 3 subtypes (Figure 6C). The immune stimulating factor box plot showed significant differences in the expression of *CD40, CXCR4, ICOS* and *MICB* among 3 subtypes (Kruskal Wallis test, *p* < 0.05) (Figure 6D). Collectively, three stable PD subtypes (C1, C2 and C3) were identified and the key differences included: C1 had the highest diagnostic risk score; C2 had the highest immune score; C1–C3 showed decreasing ESTIMATE/matrix scores and increasing tumor purity. These subtypes also differed in immune cell infiltration (T cells CD8, activated NK cells), drug sensitivity, and expression of chemokines (CCL5, CCL7), receptors (CCR4, CXCR2) and immune factors (CD40, CD96). These results highlight the heterogeneity of PD peripheral immune features and support subtype‐specific treatment strategies.

### 3.6 Functional Enrichment Analysis of PD Subtypes

We conducted differential expression analysis on the gene expression matrix by using R package “limma” and obtained a total of 443 DEGs between C2 and C1 group, with 189 upregulated genes and 254 downregulated genes; a total of 569 DEGs between C3 and C1 group, with 222 upregulated genes and 347 downregulated genes; a total of 48 DEGs between C3 and C2 group, with 23 upregulated genes and 25 downregulated genes (Supporting Information Dataset 3).

**FIGURE 7:**
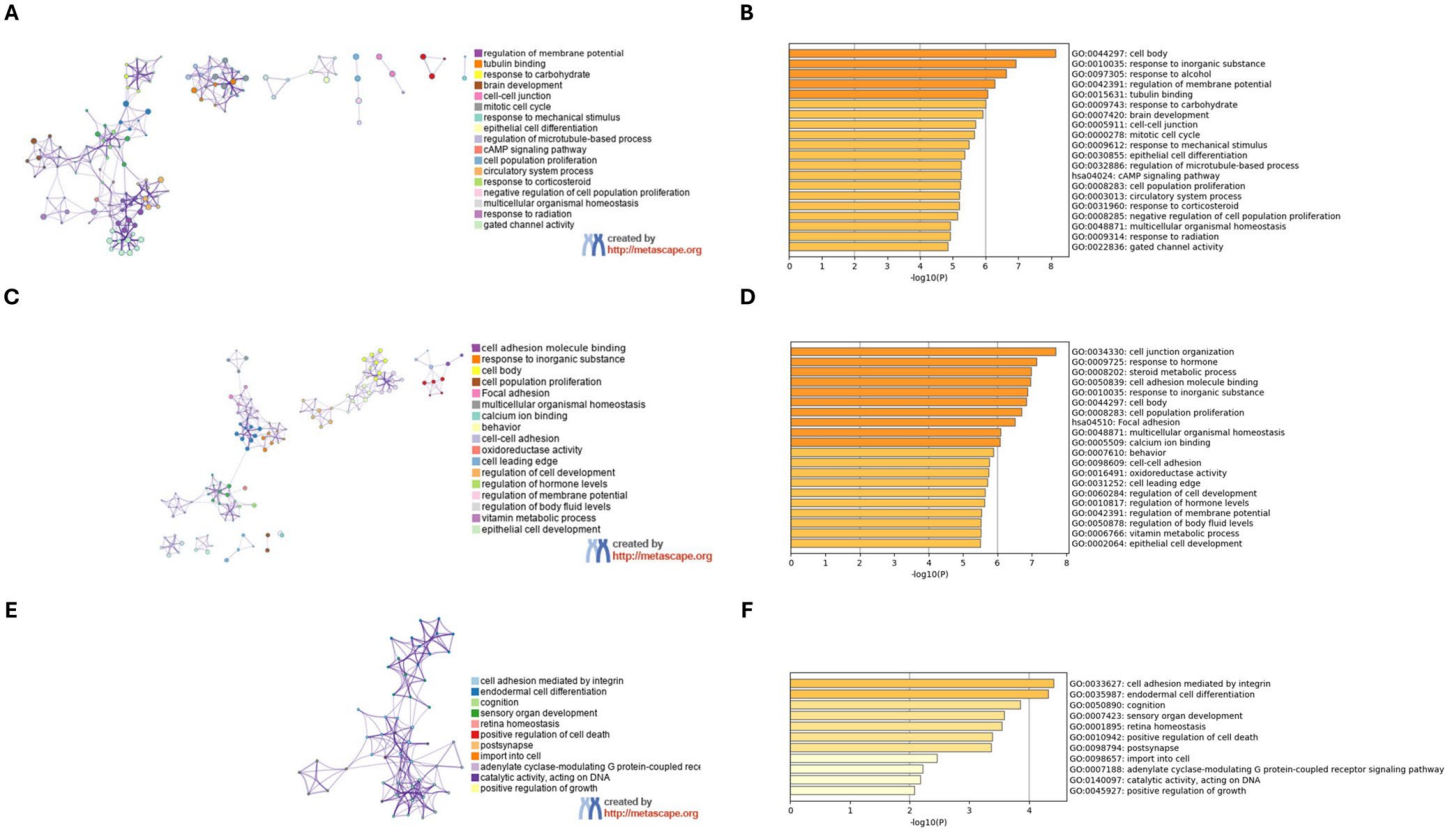
GO/KEGG functional enrichment analysis. (A) A network diagram of the top enrichment function in the C2–C1 group; (B) The bar plot of C2–C1 group with top enrichment function is represented by *p* value for color. The darker color indicated the more significant *p* value; (C) Network diagram of top enrichment function in C3–C1 group; (D) Bar plot of top enriched function in C3–C1 group; (E) Network diagram of top enrichment function in C3–C2 group; (F) Bar plot with C3–C2 group top enrichment function. GO, Gene Ontology; KEGG, Kyoto Encyclopedia of Genes and Genomes.

**FIGURE 8:**
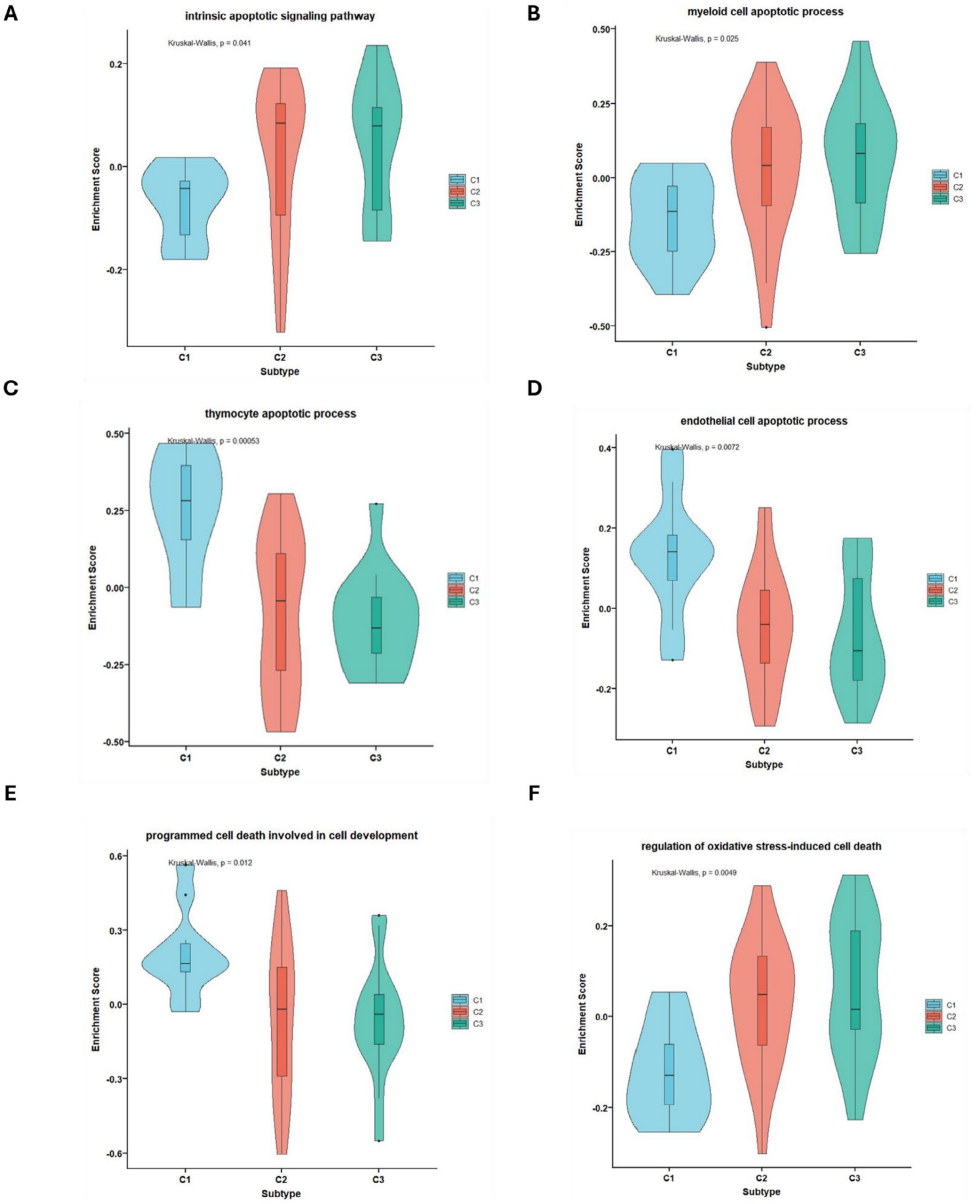
The ssGSEA of 3 subtypes. The Enrichment Fraction Violin Chart of (A) Intrinsic apoptotic signaling pathway; (B) Myeloid cell apoptotic process; (C) Thymocyte apoptotic Process; (D) Endothelial Cell Apoptotic Process; (E) Programmed cell death involved in cell development; (F) Regulation of oxidative stress—induced cell death. SsGSEA, single sample gene set enrich analysis.

The results of functional enrichment analysis showed that DEGs of C2‐C1 groups were mainly enriched in “cell body”, “response to inorganic tolerance”, “brain development”, “cell population promotion”, “cAMP signaling pathway”, “negative regulation of cell population promotion” and “mitotic cell cycle”, etc. (Figure 7A,B); The DEGs of C3‐C1 groups were mainly enriched in “cell population promotion”, “focal adhesions, behavior, regulation of cell development”, “steroid metabolic process”, “epiphytic cell development”, “vitamin metal process”, “calculation ion binding” and “cell adhesion molecule binding”, etc. (Figure 7C,D); The DEGs of C3‐C2 groups were mainly enriched in “positive regulation of growth”, “positive regulation of cell death”, “terminal cell differentiation”, “recurrent homeostasis”, “adenylate cyclization modulating G protein coupled receptor signaling pathway” and “catalytic activity, and action on DNA”, etc. (Figure 7E,F). The details of the above GO and KEGG functional enrichment were summarized in the Supporting Information Dataset 4. The results of ssGSEA enrichment analysis showed significant differences in the enrichment scores of cell death related functions among 3 subtypes, such as “intrinsic apoptotic signaling pathway”, “myoid cell apoptotic process”, “chromosomal apoptotic process”, “endometrial cell apoptotic process”, “programmed cell death involved in cell development” and “regulation of oxidative stress‐induced cell death” (Figure 8). DEGs among these 3 subtypes were enriched in distinct biological processes and pathways: C2 versus C1 DEGs enriched on brain development and cell cycle; C3 versus C1 DEGs on cell adhesion and metabolic processes; C3 versus C2 DEGs on cell growth and death regulation. ssGSEA revealed subtype‐specific differences in cell death‐related pathways (e.g., apoptotic signaling, oxidative stress‐induced cell death), further confirming functional heterogeneity among these 3 subtypes.

### 3.7 PD Peripheral Blood Single Cell Data Processing, Clustering and Annotation

**FIGURE 9:**
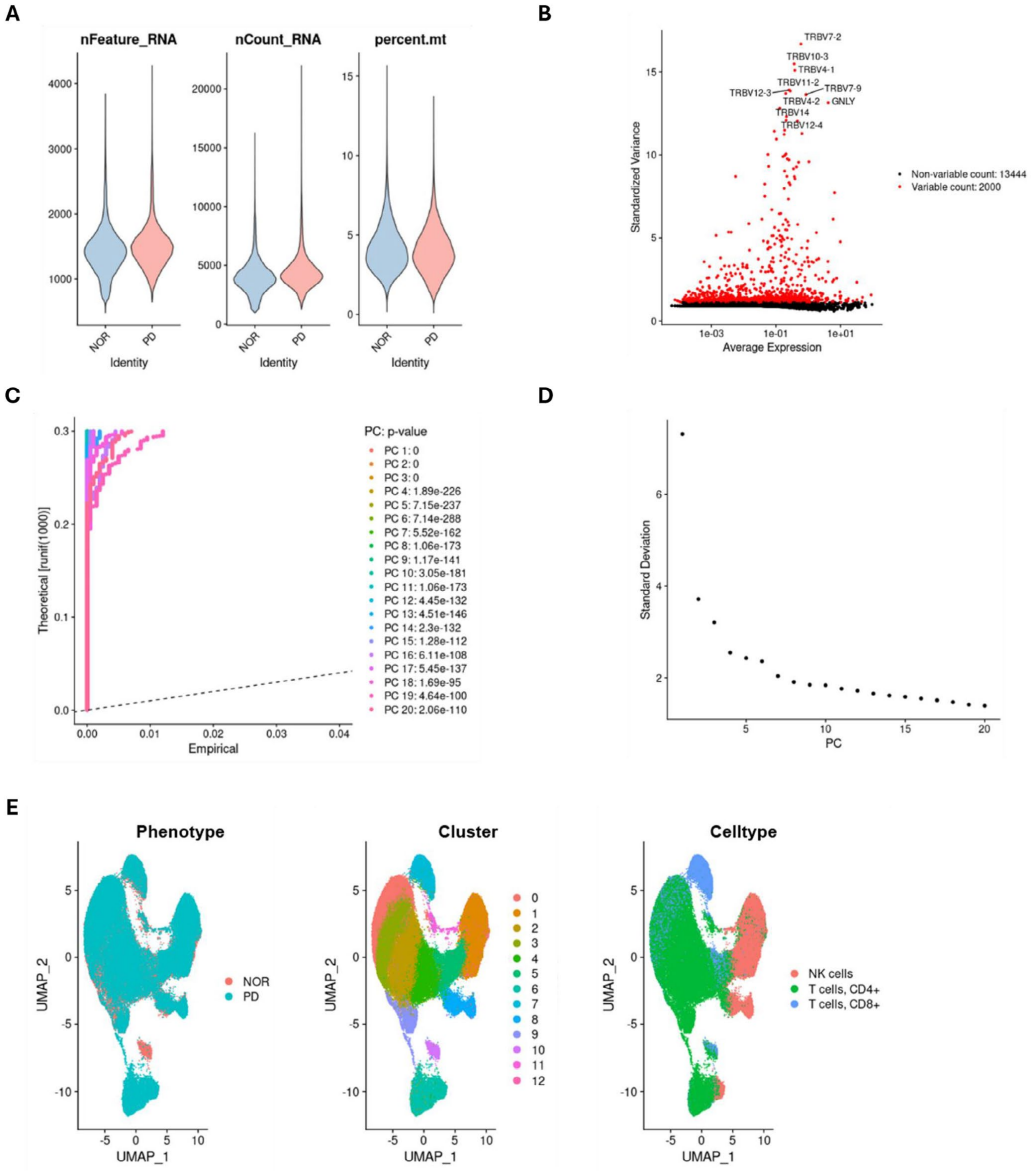
The single cell data processing, clustering and annotation. (A) Quality control of single cell count data; (B) The top 2000 genes with high variability between cells were identified, and the top 10 genes with the highest frequency of variation were labeled; (C) JackStrawPlot of tshe top 20 PCA principal components; (D) ElbowPlot of the top 20 PCA principal components; (E) The cell clustering obtained by the UMAP algorithm. PCA, principal component analysis; UMAP, uniform manifold approval and projection.

We filtered out cells and genes with poor quality and finally obtained 89 162 cells and 15 444 genes from scRNA data. The violin chart shows the gene, count and mitochondrial gene proportion of the single‐cell data, demonstrating good data quality control (Figure 9A). We normalized the counts using the “LogNormalize” method by default, which divides the counts of each gene by the total count of cells, multiplied by 10 000, and then performs logarithmic conversion. The “FindVariableFeature” function was used to select genes with high variability between cells for subsequent analysis (Figure 9B). The PCA analysis was performed, and the final number of significance dimensions was determined, with the best 10 dimensions selected for subsequent analysis (Figure 9C,D). Furthermore, the UMAP algorithm was used to explore cell clustering based on the optimal dimensions, and finally we got 12 cell clusters. These cell clusters were annotated, and three cell types including *NK cells*, *T cells CD4^+^* and *T cells CD8^+^* were obtained (Figure 9E). After quality control, 89 162 cells and 15 444 genes were retained, and 12 cell clusters were annotated into three immune cell types (*NK cells*, *T cells CD4* ^+^ and *T cells CD8* ^+^). Notably, since *macrophage* and *B‐cell* populations were not identified with sufficient confidence in this single‐cell dataset, the scRNA‐seq analysis was not used to validate the CIBERSORT‐derived *macrophage*‐like or *B‐cell* signatures. Instead, it was used to explore ferroptosis‐related transcriptional features in the annotated peripheral *NK,* *CD4* ^+^ *T‐cell* and *CD8* ^+^ *T‐cell* populations.

### 3.8 Ferroptosis‐Related Transcriptional Features in Peripheral Immune Cell Subsets

**FIGURE 10:**
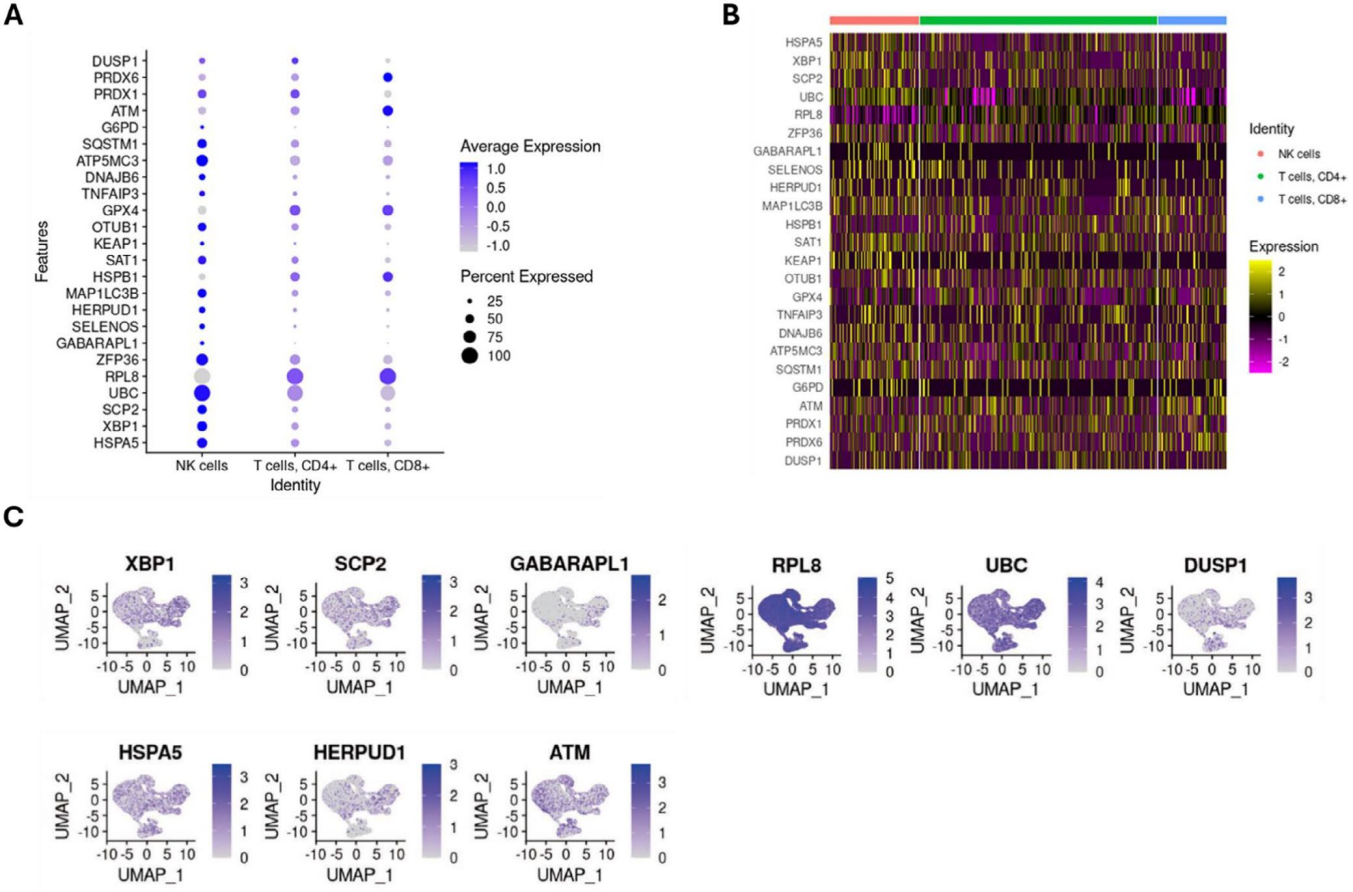
The identification of CFGs. (A) Dot plot map revealed the expression features of 24 CFGs. (B) Heat map of the expression of 24 CFGs in CD4^+^ T cell, CD8^+^ T cell and NK cell; (C) Feature plot of 9 key CFGs including XBP1, SCP2, GABA, RAPL1, RPL8, UBC, DUSP1, HSPA5, HERPUD1 and ATM. CFGs, ferroptosis characteristic genes.

**FIGURE 11:**
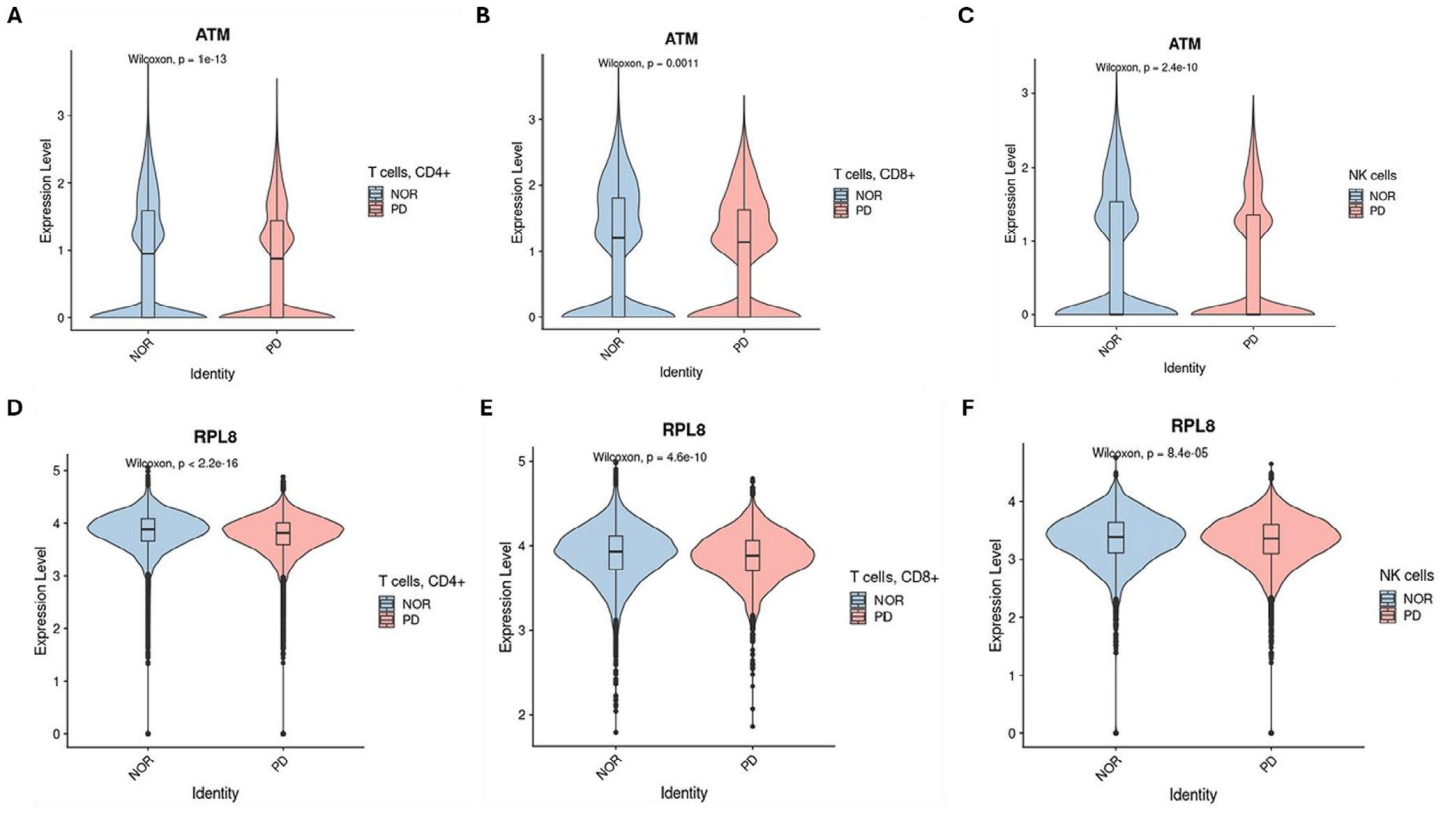
The significantly different expression levels of ATM and RPL8 in CD4^+^ T cell, CD8^+^ T cell and NK cells between PD and control groups. (A‐C) ATM, ATM Serine/Threonine Kinase. (D‐F) RPL8, Ribosomal Protein L8.

**FIGURE 12:**
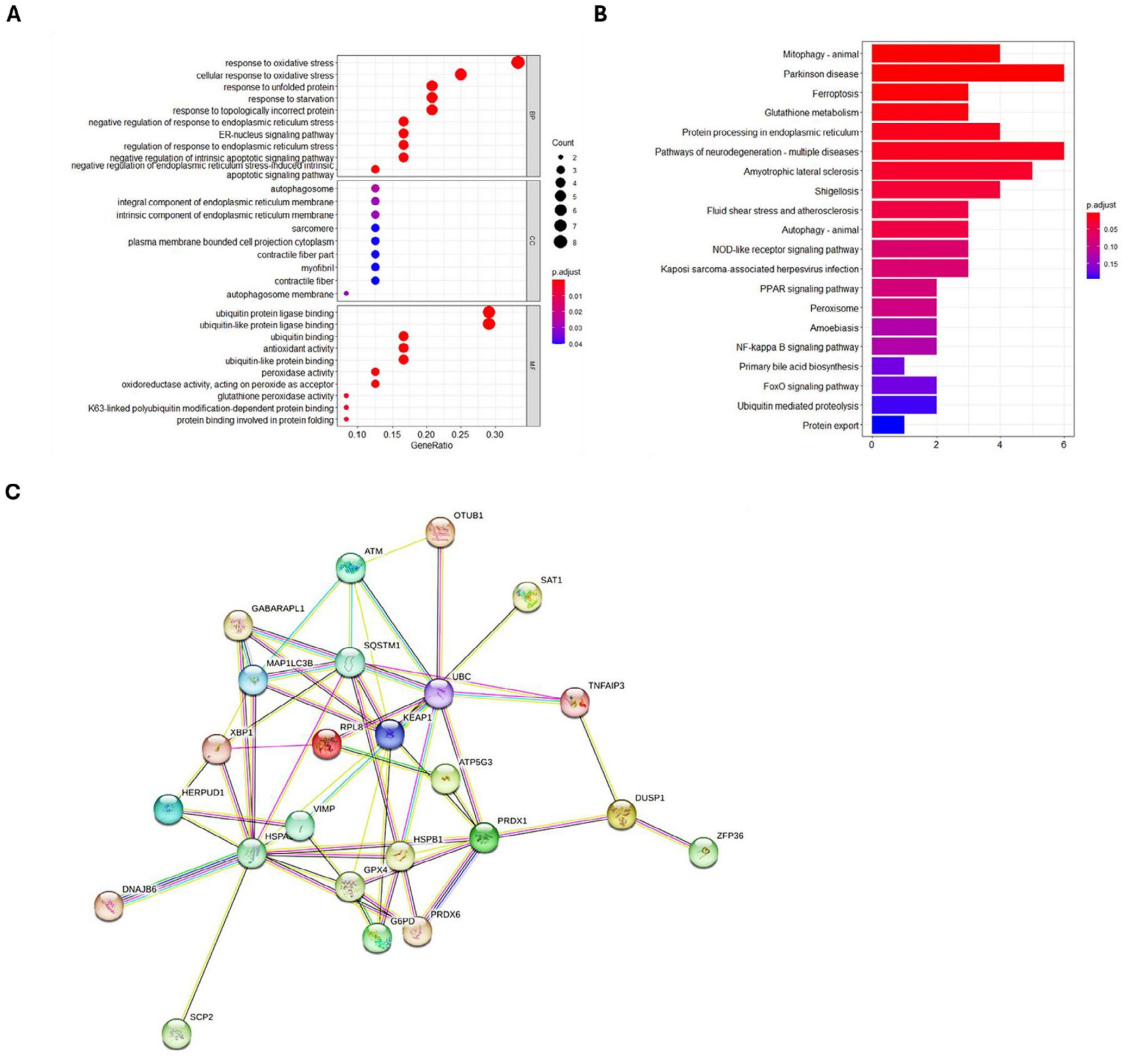
The functional enrichment analysis of cell‐characterized ferroptosis genes based on the PD single‐cell sequencing dataset. (A) GO enrichment of ferroptosis genes in binding protein (BP), cell component (CC), molecular function (MF). (B) KEGG enrichment of these ferroptosis genes in different signaling pathways. (C) PPI‐network analysis of these ferroptosis gene.

We further analyzed the differential expression genes in each cell type, screened the specific ferroptosis factors, and finally obtained 24 characteristic ferroptosis genes (CFGs). The dot‐plot visualized the labeled CFGs among each cell type (Figure 10A). The thermogram revealed the expression level of the CFGs in different cell types (Figure 10B). Feature Plot showed the expression distribution of 9 CFGs with the largest difference among these three cell types, which were *XBP1, SCP2, GABARAPL1, RPL8, UBC, DUSP1, HSPA5, HERPUD1* and *ATM* (Figure 10C). Among them, *RPL8* and *ATM* were significantly different in *NK cells*, *T cells CD4^+^* and *T cells CD8^+^* between normal and PD groups (Wilcoxon Rank Sum Test, *p* < 0.05) (Figure 11). The function enrichment analysis on 24 CFGs showed that these genes were mainly enriched in “ER‐nucleus signaling pathway”, “response to oxidative stress”, “cellular response to oxidative stress”, “negative regulation of response to endogenous reticulum stress”, “negative regulation of endogenous reticulum stress induced intrinsic apoptotic signaling pathway”, “negative regulation of intrinsic apoptotic signaling pathway” and “autophagosome”, etc. (Figure 12A,B, Table 4 and Supporting Information Dataset 5). The PPI network analysis of these genes showed that *KEAP1, SQSTM1, MAP1LC3B* and *HSPA5* interacted strongly with other genes (Figure 12C). In summary, 24 CFGs were identified with 9 key CFGs showing the most significant differences (*XBP1, SCP2, GABARAPL1, RPL8, UBC, DUSP1, HSPA5, HERPUD1* and *ATM*). *RPL8* and *ATM* were differentially expressed in *NK cells, T cells CD4* ^+^ and *T cells CD8* ^+^ between PD and normal groups. CFGs were enriched in oxidative stress response, ferroptosis and the PD‐related pathways, and the PPI network analysis highlighted key interacting genes (*KEAP1, SQSTM1, MAP1LC3B* and *HSPA5*), indicating their vital roles in coordinating ferroptosis and immune regulation.

**TABLE 4:** The top enrichment functions of 24 CFGs obtained from GO/KEGG analysis.

Category	GO	Description	Count	*p*.adjust
GO biological processes	GO:0006979	Response to oxidative stress	8	7.85E‐05
	GO:1903573	Negative regulation of response to endoplasmic reticulum stress	4	0.000152
	GO:0006984	ER‐nucleus signaling pathway	4	0.000152
	GO:0034599	Cellular response to oxidative stress	6	0.000577
	GO:1902236	Negative regulation of endoplasmic reticulum stress‐induced intrinsic apoptotic signaling pathway	3	0.000577
	GO:0006986	Response to unfolded protein	5	0.000593
	GO:1905897	Regulation of response to endoplasmic reticulum stress	4	0.000664
	GO:0042594	Response to starvation	5	0.000664
	GO:0035966	Response to topologically incorrect protein	5	0.000721
	GO:2001243	Negative regulation of intrinsic apoptotic signaling pathway	4	0.000936
GO cellular components	GO:0005776	Autophagosome	3	0.024876
	GO:0030176	Integral component of endoplasmic reticulum membrane	3	0.029348
	GO:0000421	Autophagosome membrane	2	0.029348
	GO:0031227	Intrinsic component of endoplasmic reticulum membrane	3	0.029348
	GO:0030017	Sarcomere	3	0.039895
	GO:0032838	Plasma membrane bounded cell projection cytoplasm	3	0.039895
	GO:0044449	Contractile fiber part	3	0.039895
	GO:0030016	Myofibril	3	0.039895
	GO:0043292	Contractile fiber	3	0.040129
GO molecular functions	GO:0031625	Ubiquitin protein ligase binding	7	7.95E‐06
	GO:0044389	Ubiquitin‐like protein ligase binding	7	7.95E‐06
	GO:0043130	Ubiquitin binding	4	0.000137
	GO:0016209	Antioxidant activity	4	0.000168
	GO:0032182	Ubiquitin‐like protein binding	4	0.000208
	GO:0004601	Peroxidase activity	3	0.001012
	GO:0016684	Oxidoreductase activity, acting on peroxide as acceptor	3	0.001084
	GO:0004602	Glutathione peroxidase activity	2	0.005304
	GO:0070530	K63‐linked polyubiquitin modification‐dependent protein binding	2	0.005304
	GO:0044183	Protein binding involved in protein folding	2	0.005741
KEGG pathway	hsa04137	Mitophagy—animal	4	0.001716
	hsa05012	Parkinson disease	6	0.001716
	hsa04216	Ferroptosis	3	0.003617
	hsa00480	Glutathione metabolism	3	0.007241
	hsa04141	Protein processing in endoplasmic reticulum	4	0.012908
	hsa05022	Pathways of neurodegeneration—multiple diseases	6	0.013433
	hsa05014	Amyotrophic lateral sclerosis	5	0.02186
	hsa05131	Shigellosis	4	0.031047
	hsa05418	Fluid shear stress and atherosclerosis	3	0.039131
	hsa04140	Autophagy—animal	3	0.039131

### 3.9 Laboratory Validation of Key Characteristic Ferroptosis Genes

**FIGURE 13:**
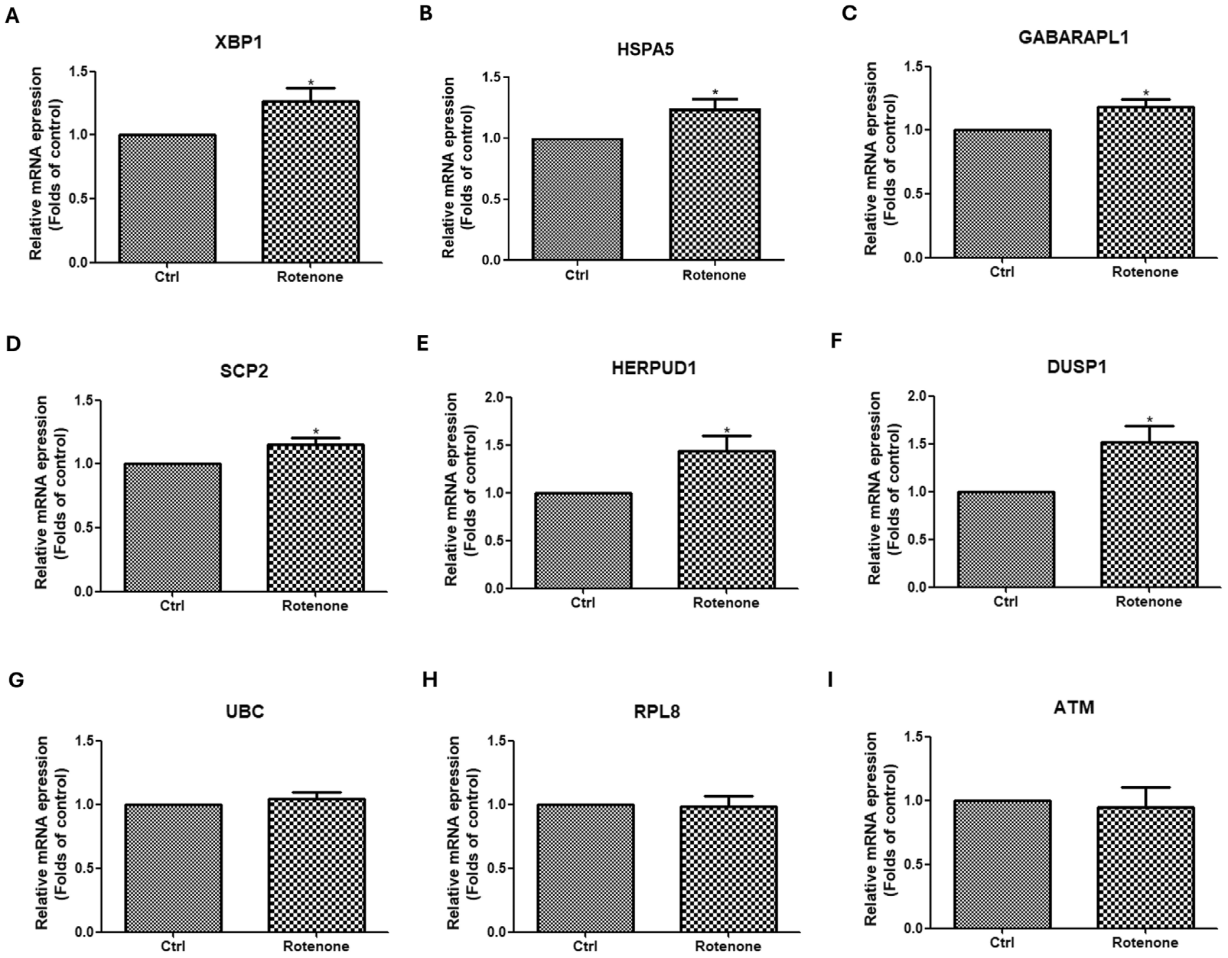
The validation of the mRNA expression of the 9 key CFGs. (A) XBP1, X‐box binding protein 1; (B) HSPA5, Heat Shock Protein Family A Member 5; (C) GABARAPL1, GABA Type A Receptor Associated Protein Like 1; (D) SCP2, Sterol Carrier Protein 2; (E) HERPUD1, Homocysteine‐inducible, Endoplasmic reticulum stress‐inducible, Ubiquitin‐like domain member 1; (F) DUSP1, Dual Specificity Phosphatase 1; (G) UBC, Ubiquitin C; (H) RPL8, Ribosomal Protein L8; (I) ATM, Ataxia Telangiectasia‐mutated Gene. *n* = 5. *p < 0.05 versus Control group.

SH‐SY5Y human neuroblastoma cells treated with rotenone have been widely used to construct in vitro models of PD [44, 45]. In this study, rotenone was also used to induce neuron injury and mimic PD in SH‐SY5Y cells. We utilized the qPCR method to measure the relative expression levels of above 9 key CFGs in both rotenone‐treated cells and control cells. We found that the expression of *XBP1, SCP2, GABARAPL1, DUSP1, HSPA5* and *HERPUD1* in the PD cell model significantly increased compared to the control group (*p* < 0.05), whereas the expression of *UBC, RPL8* and *ATM* showed no significant difference (*p* > 0.05) (Figure 13). The significant upregulated expression of *XBP1, SCP2, GABARAPL1, DUSP1, HSPA5* and *HERPUD1* in the PD cell model that is consistent with findings from transcriptomic analysis. No significant differences of *UBC, RPL8* and *ATM* expressions were observed, which is possibly due to cell‐type‐specific regulatory differences (immune cells vs. neuronal cells). These results confirm the involvement of CFGs in PD‐related neuronal stress responses.

## 4 Discussion

PD is becoming a common disease in the middle‐aged and elderly. In recent years, the clinical prevalence and disability rate of PD remains high, causing serious impacts on patients' physical and mental health, family economy, and quality of life [46]. The main reason is the unclear pathogenesis of PD and lack of effective treatment approach. In this study, we suggest that the mechanisms of ferroptosis, such as the imbalance of iron ion metabolism, oxidative stress and glutamate metabolism, have much in common with the pathogenesis of PD. Ferroptosis might promotes the development of PD and targeting ferroptosis is promising for PD therapy [47]. But its specific signaling pathway and mechanism still need further exploration. Although PD pathology is centered in the substantia nigra, peripheral blood transcriptomic profiles may capture systemic immune activation, oxidative stress, and ferroptosis‐related responses that accompany with PD. In this study, blood‐derived AEFRGs were interpreted as accessible peripheral signatures rather than direct measurements of brain pathology. The substantia nigra dataset was used only for validation, and the observed performance in this dataset should be viewed as supportive cross‐tissue evidence, not as proof of a direct blood–brain correlation. Potential biases include tissue‐specific gene expression, differences in cell composition, postmortem brain tissue effects, disease stage, medication status and platform heterogeneity.

### 4.1 Iron Homeostasis and Lipid Peroxidation

In our study, we collated the transcriptome datasets in GEO database. We obtained the ferroptosis gene set from the existing database and compared the expression of FRGs between the PD and normal groups, and a total of 16 AEFRGs were extracted. Chromosome mapping showed that most of these genes were distributed on different chromosomes, which indicates that the biological functions and processes involved in these genes might be different. The dysregulation of gene expression that involved in maintaining iron homeostasis could lead to the abnormal iron content and ferroptosis in PD patients, which ultimately results in degenerative changes in patients' CNS [48]. Among these 16 AEFRGs, *ELAVL1* encodes an RNA binding protein, while *MYB* is a transcription factor, both of which are associated with iron transport, storage and uptake pathways. Lin Z et al. found that promoting the cytoplasmic distribution of *ELAVL1* increased the accumulation of *SLC7A11* and the level of glutathione (GSH) in gastric cancer cells, consequently inhibiting the accumulation of iron and preventing ferroptosis [49]. Ferroptosis cell releases more iron into the intercellular space along with cell lysis, which forms a high iron microenvironment, causes the accumulation of iron in the surrounding neurons, and further induces more cells undergoing ferroptosis. These processes also show the continuous disease progression of PD patients [50].

Lipid peroxide, which is produced by abnormal lipid metabolism, is one of the fundamental pathological mechanisms of ferroptosis. Lipid peroxide could directly promote neurodegenerative changes in the substantia nigra. The reduced synthesis of GSH is the core of amino acid metabolic pathway regulating ferroptosis. Oxidative stress‐caused insufficient intracellular GSH leads to declined cell capability of GSH‐dependent clearance of lipid peroxide [51]. Among these 16 AEFRGs, *GPX2* is a GSH peroxidase, and *PTGS2* could act as both a double oxygenase and peroxidase. Du H et al. observed an increase of *GPX2* expression in lung adenocarcinoma, which increased glucose uptake and ATP production, reduced GSH expression, promoted the production of ROS, and induced cell ferroptosis [52]. In this study, increased expressions of *GPX2* and *PTGS2* were also observed in PD patients, which is consistent with the previous study. We also observed high expression of genes *ALOX5* and *NOX3*, which are related to lipid oxidation and, in particular, *NOX3* encodes an oxidase with the ability to produce peroxides and reactive oxygen species [53].

We conducted functional enrichment analysis on these 16 AEFRGs and found that these genes are involved in regulating cell death, cell stress response, cell catabolism, neuron death and neurodegenerative diseases, which provides evidence for the involvement of ferroptosis in the pathogenesis of PD. We further analyzed the interaction between these AEFRGs and miRNAs, TFs or RBPs, and found that these 16 AEFRGs interacted with 318 miRNAs, 58 TFs and 198 RBPs. All these small molecules play important roles in regulating iron accumulation and maintaining iron homeostasis.

### 4.2 Neuroinflammation and PD Diagnostic Model

Li et al. observed reduced B lymphocyte proliferation and lower regulatory B lymphocyte (transitional B lymphocyte) proportions in PD patients versus controls, accompanied by an increased fraction of pro‐inflammatory cytokine‐producing B lymphocytes [54]. Macrophages can be divided into M1 (pro‐inflammatory) and M2 (counter‐inflammatory) subtypes, PD is pathologically characterized by neuroinflammation, which is less amount of anti‐inflammatory macrophages and more pro‐inflammatory macrophages [55].

Neuroinflammation plays a major role in the pathogenesis of PD and leads to the selective injury of dopaminergic neurons. Therefore, immune cells and inflammatory molecules are involved in the occurrence and development of PD, and ferroptosis genes might promote the progress of disease by regulating peripheral immune cell homeostasis and the crosstalk between peripheral and central immune systems. In this study, we analyzed the correlation between 16 AEFRGs and immune cells and found that *PTGS2* and *ALOX5* genes were correlated with specific immune cells, such as neutrophils. In the comparison of immune cell infiltration between PD and normal samples, we found that macrophages had a lower degree of infiltration in PD samples, while B cells had a higher degree. The high expression of Toll like receptors in B cells can promote the release of anti‐inflammatory cytokines, ultimately inducing adaptive immune development in T cells [56]. Ferroptosis gene induces the mature of specific peripheral immune cells and regulates the release of proinflammatory factors, which affects the integrity of the blood–brain barrier and indirectly contributes to neuronal damage [57].

We attempted to identify markers related to PD diagnosis using these 16 AEFRGs and found that except for *NOX3*, all other 15 genes belong to diagnostic markers. The diagnostic model established based on these 15 genes has good diagnostic ability and application value. In Xing N et al.'s study, the combination of ferroptosis genes and immune genes was used to establish a diagnostic model of PD, and the AUC of the diagnostic model was 0.831 [58]. However, in this study, the AUC of the diagnostic model exceeded 0.9 in all the three datasets. Our high accuracy might be due to more FRGs were included in our model.

Because ferroptosis is involved in a variety of different signal pathways, clustering the samples using AEFRGs could identify the molecular subtypes of patients, which is more conducive to the implementation of personalized treatment programs for patients. In this study, 276 FRGs were used to cluster the PD samples, and we found that the samples can be stably grouped into three categories (C1, C2 and C3). There are differences between the diagnostic model scores, immune scores, drug sensitivity scores, immune infiltration, immunosuppressive factors, chemokines and gene function enrichment of these categories. These results suggest that appropriate treatment plans could be selected based on the different molecular subtypes of PD patients.

PD is characterized by the core pathological feature of damage to central dopaminergic neurons, but it is accompanied by systemic immune disorders, iron homeostasis imbalance, and a systemic cascade reaction of neuroinflammation [59]. The activation of microglia in the brain interacts closely with the peripheral immune system. Peripheral immune cells (such as T cells and NK cells, which are the focus of this study) can indirectly reflect the immune microenvironment state in the brain by influencing the function of the blood–brain barrier. FRGs can regulate the maturation of immune cells and the release of pro‐inflammatory factors [60], and their peripheral expression characteristics can reveal the systemic metabolic and immune regulatory background related to PD. Therefore, the analysis results based on the peripheral blood datasets in this study can indirectly reflect the systemic molecular changes that are related to brain pathology, providing a reference for the pathological mechanism and diagnosis of PD.

We further analyzed the scRNA data derived from peripheral blood and identified three major immune cell types: NK cells, CD4^+^ T cells and CD8^+^ T cells. The expression characteristics of FRGs in these peripheral immune cells (e.g., RPL8 and ATM) primarily reflect PD‐associated peripheral immune regulation status, rather than direct changes in CNS‐resident immune cells [61]. Ferroptosis is closely associated with both endoplasmic reticulum stress (ERS) and oxidative stress. Notably, ERS modulates ferroptosis, particularly through the protein kinase R‐like ER kinase (PERK)–activating transcription factor 4 (ATF4) signaling axis, and accumulating evidence has further highlighted a tight link between ferroptosis and oxidative stress [62]. It should be noted that some genes classified as ferroptosis‐related (e.g., HSPA5 and XBP1) are also involved in multiple stress pathways, such as ERS, oxidative stress and unfolded protein response. Their increased expression is more likely to represent the stress adaptation state of immune cells in the disease context. The changes of these molecules provide clues for understanding the PD‐related systemic immune response and its potential link with cell death regulation [63]. Consistent with previous studies, peripheral immune cell dysfunction can indirectly modulate CNS pathology through the peripheral‐central immune crosstalk. Activated peripheral T cells can secrete inflammatory cytokines to alter blood–brain barrier permeability, while NK cells may participate in the clearance of pathological substances in the periphery, thereby reducing their potential neurotoxicity [64]. We explored the pathways of differential gene enrichment in three kinds of cells, and found that these genes were enriched in oxidative stress, mitochondrial autophagy, PD, ferroptosis and other pathways, which further explained the vital role of ferroptosis in immune cell infiltration of PD.

### 4.3 Verification of 9 Key CFGs

In the qPCR verification of this study, rotenone‐treated SH‐SY5Y cells were used as an in vitro PD model. This model can effectively simulate mitochondrial dysfunction and oxidative stress conditions that mimic PD, but apart from induction of neurotoxicity and ferroptosis [65], but it also activates various cellular stress pathways, such as ERS, unfolded protein response and apoptosis‐related pathways [66]. HSPA5 and XBP1 are closely related to ERS and oxidative stress, both of which are strongly associated with ferroptosis [67, 68]. Therefore, the observed changes in the expression of FRGs in this model may be influenced by non‐ferroptosis stress pathways, and the results are more suitable for understanding the involvement of the ferroptosis‐related stress network rather than a strict specificity verification of the ferroptosis process. This characteristic of the model limits the single interpretation of qPCR results regarding the molecular mechanism of ferroptosis, and further differentiation is needed in subsequent studies by combining more direct ferroptosis functional indicators, such as lipid ROS, iron loading and ferrostatin‐1 rescue. qPCR validation showed significant upregulation of XBP1, SCP2, GABARAPL1, DUSP1, HSPA5 and HERPUD1 in the PD cell model, which is consistent with the findings from transcriptomic analysis. These results confirm the involvement of CFGs in PD‐related neuronal stress responses. RPL8 and ATM failed to reproduce the single‐cell analysis results in the in vitro model, this may be related to the differences between in vivo and in vitro models, the composition of cell types, and the different regulatory levels.

### 4.4 Limitations

There are still some limitations in this study. First, although we have found the markers of ferroptosis in PD, these genes might participate in multiple pathways, for example, oxidative stress and autophagy, thus mediating the occurrence and progression of PD with unidentified mechanisms. Second, we established a diagnostic model using 15 key genes, but it must be acknowledged that, despite its high accuracy, its practical application value is still limited due to the large number of genes that need to be detected. Additional external validation, and/or include cross‐validation performance metrics, such as calibration plots or decision curve analysis, will improve its confidence. Third, although we concluded that ferroptosis genes induce immune infiltration and differentiation through correlation analysis, immune infiltration analysis and Single‐cell sequencing analysis, the specific signal pathway still deserves further analysis and clarify. Fourth, the ferroptosis is not the only cause of PD, which needs further correlated investigation with other pathological processes, for example, mitochondrial dysfunction, oxidative stress, neuroinflammation and neuronal toxicity. Furthermore, in this study, peripheral blood transcriptome data was used in the discovery stage for ferroptosis‐related molecular features, while the substantia nigra tissue data was mainly used for independent validation of the model and confirmed whether the results of peripheral blood transcriptome data analysis reflect the brain state. This strategy is based on the comprehensive consideration of both scientific and translational angles. However, although the organ‐peripheral blood connection does exist, the pathological condition of brain could not be acurrately reflected by peripheral blood. Because all the organs exchange metabolic substrates and immune cells with peripheral blood, which accounts for the complexity of blood content, and there is a blood‐brain barrier that limits these exchanges with brain specificity [69]. According to the previous study, the expression characteristics of FRGs in the blood can serve as a systemic biomarker reflecting the overall pathological state of PD, especially related to neuroinflammation and the vulnerability of neurons [70]. These peripheral features are highly correlated with the brain pathology (verified through the substantia nigra tissue dataset), suggesting that they jointly participate in a systemic disease network. So, this study relied on the markers derived from peripheral blood for analysis, the scRNA‐seq dataset is also derived from peripheral blood (not substantia nigra), which has inherent limitations and should be further verified in the future.

## 5 Conclusions

In conclusion, ferroptosis is involved in the progression of PD, and ferroptosis‐related transcriptional signatures may reflect peripheral immune dysregulation and provide candidate diagnostic markers for PD.

## Author Contributions

Lei Cheng, Jing Zhang and Zhong‐Yan Zhou wrote the manuscript. Wen‐Ting Shi and Jing Zhang conducted the experiment and analyzed the data. Qing Ye collected the experimental protocols. Cheng Huang designed the study. Chen Huang and Zhong‐Yan Zhou revised and submitted the manuscript.

## Funding

This study was supported by the grant from the Macao Science and Technology Development Fund (002/2023/ALC, 0009/2025/RDP), The Science and Technology Development Fund, Macau SAR (File no. 006/2023/SKL; File no. 0003/2025/NRP), Shanghai Human Resources and Social Security Bureau (Pujiang Project, 2023PJD094), Shanghai Commission of Science and Technology (22ZR1462200), Health Commission of Shanghai (GWIV‐28) and The University of Hong Kong.

## Conflicts of Interest

The authors declare no conflicts of interest.

## Supporting information


**Figure S1:** Chromosome location of the genes.

Dataset 1.

Dataset 2.

Dataset 3.

Dataset 4.

Dataset 5.

## Data Availability

The data that support the findings of this study are available in the Materials and Methods, Results, and Supporting Information of this article.

## References

[1] E. Tolosa , A. Garrido , S. W. Scholz , and W. Poewe , “Challenges in the Diagnosis of Parkinson's Disease,” Lancet Neurology 20 (2021): 385–397. 33894193 10.1016/S1474-4422(21)00030-2 PMC8185633

[2] M. T. Hayes , “Parkinson's Disease and Parkinsonism,” American Journal of Medicine 132 (2019): 802–807. 30890425 10.1016/j.amjmed.2019.03.001

[3] S. G. Reich and J. M. Savitt , “Parkinson's Disease,” Medical Clinics of North America 103 (2019): 337–350. 30704685 10.1016/j.mcna.2018.10.014

[4] O. B. Tysnes and A. Storstein , “Epidemiology of Parkinson's Disease,” Journal of Neural Transmission (Vienna) 124 (2017): 901–905. 10.1007/s00702-017-1686-y 28150045

[5] R. B. Schneider , J. Iourinets , and I. H. Richard , “Parkinson's Disease Psychosis: Presentation, Diagnosis and Management,” Neurodegenerative Disease Management 7 (2017): 365–376. 29160144 10.2217/nmt-2017-0028

[6] C. Lei , Z. Zhongyan , S. Wenting , et al., “Identification of Necroptosis‐Related Genes in Parkinson's Disease by Integrated Bioinformatics Analysis and Experimental Validation,” Frontiers in Neuroscience 17 (2023): 1097293. 37284660 10.3389/fnins.2023.1097293 PMC10239842

[7] X. Dong‐Chen , C. Yong , X. Yang , S. Chen‐Yu , and P. Li‐Hua , “Signaling Pathways in Parkinson's Disease: Molecular Mechanisms and Therapeutic Interventions,” Signal Transduction and Targeted Therapy 8 (2023): 73. 36810524 10.1038/s41392-023-01353-3 PMC9944326

[8] Q. Zhang , H. Zhao , M. Luo , et al., “The Classification and Prediction of Ferroptosis‐Related Genes in ALS: A Pilot Study,” Frontiers in Genetics 13 (2022): 919188. 35873477 10.3389/fgene.2022.919188 PMC9305067

[9] Y. Wang , B. Tang , J. Zhu , et al., “Emerging Mechanisms and Targeted Therapy of Ferroptosis in Neurological Diseases and Neuro‐Oncology,” International Journal of Biological Sciences 18 (2022): 4260–4274. 35844784 10.7150/ijbs.72251 PMC9274504

[10] H. Jiang , J. Wang , J. Rogers , and J. Xie , “Brain Iron Metabolism Dysfunction in Parkinson's Disease,” Molecular Neurobiology 54 (2017): 3078–3101. 27039308 10.1007/s12035-016-9879-1

[11] D. T. Dexter , C. J. Carter , F. R. Wells , et al., “Basal Lipid Peroxidation in Substantia Nigra Is Increased in Parkinson's Disease,” Journal of Neurochemistry 52 (1989): 381–389. 2911023 10.1111/j.1471-4159.1989.tb09133.x

[12] S. Davis and P. S. Meltzer , “GEOquery: A Bridge Between the Gene Expression Omnibus (GEO) and BioConductor,” Bioinformatics 23 (2007): 1846–1847. 17496320 10.1093/bioinformatics/btm254

[13] C. R. Scherzer , A. C. Eklund , L. J. Morse , et al., “Molecular Markers of Early Parkinson's Disease Based on Gene Expression in Blood,” Proceedings of the National Academy of Sciences of the United States of America 104 (2007): 955–960. 17215369 10.1073/pnas.0610204104 PMC1766335

[14] R. Calligaris , M. Banica , P. Roncaglia , et al., “Blood Transcriptomics of Drug‐Naïve Sporadic Parkinson's Disease Patients,” BMC Genomics 16 (2015): 876. 26510930 10.1186/s12864-015-2058-3 PMC4625854

[15] T. G. Lesnick , E. J. Sorenson , J. E. Ahlskog , et al., “Beyond Parkinson Disease: Amyotrophic Lateral Sclerosis and the Axon Guidance Pathway,” PLoS One 3 (2008): e1449. 18197259 10.1371/journal.pone.0001449 PMC2175528

[16] P. Wang , L. Yao , M. Luo , et al., “Single‐Cell Transcriptome and TCR Profiling Reveal Activated and Expanded T Cell Populations in Parkinson's Disease,” Cell Discovery 7 (2021): 52. 34282123 10.1038/s41421-021-00280-3 PMC8289849

[17] M. Carlson , “hgu133a.db: Affymetrix Human Genome U133 Set Annotation Data (Chip hgu133a) (3.2.3., R. p. v., ed),” (2016).

[18] E. A. Bruford , C. R. Antonescu , A. J. Carroll , et al., “HUGO Gene Nomenclature Committee (HGNC) Recommendations for the Designation of Gene Fusions,” Leukemia 35 (2021): 3040–3043. 34615987 10.1038/s41375-021-01436-6 PMC8550944

[19] N. Zhou and J. Bao , “FerrDb: A Manually Curated Resource for Regulators and Markers of Ferroptosis and Ferroptosis‐Disease Associations,” Database: The Journal of Biological Databases and Curation 2020 (2020): 1–8. 32219413 10.1093/database/baaa021 PMC7100629

[20] H. Zhang , P. Meltzer , and S. Davis , “RCircos: An R Package for Circos 2D Track Plots,” BMC Bioinformatics 14 (2013): 244. 23937229 10.1186/1471-2105-14-244 PMC3765848

[21] Y. Zhou , B. Zhou , L. Pache , et al., “Metascape Provides a Biologist‐Oriented Resource for the Analysis of Systems‐Level Datasets,” Nature Communications 10 (2019): 1523. 10.1038/s41467-019-09234-6 PMC6447622 30944313

[22] G. Yu , “Gene Ontology Semantic Similarity Analysis Using GOSemSim,” Methods in Molecular Biology 2117 (2020): 207–215. 31960380 10.1007/978-1-0716-0301-7_11

[23] G. Zhou , O. Soufan , J. Ewald , R. E. W. Hancock , N. Basu , and J. Xia , “NetworkAnalyst 3.0: A Visual Analytics Platform for Comprehensive Gene Expression Profiling and Meta‐Analysis,” Nucleic Acids Research 47 (2019): W234–W241. 30931480 10.1093/nar/gkz240 PMC6602507

[24] J. A. Castro‐Mondragon , R. Riudavets‐Puig , I. Rauluseviciute , et al., “JASPAR 2022: The 9th Release of the Open‐Access Database of Transcription Factor Binding Profiles,” Nucleic Acids Research 50 (2022): D165–D173. 34850907 10.1093/nar/gkab1113 PMC8728201

[25] D. Karagkouni , M. D. Paraskevopoulou , S. Chatzopoulos , et al., “DIANA‐TarBase v8: A Decade‐Long Collection of Experimentally Supported miRNA‐Gene Interactions,” Nucleic Acids Research 46 (2018): D239–D245. 29156006 10.1093/nar/gkx1141 PMC5753203

[26] X. Zhou , M. Jiang , Z. Liu , et al., “Na(+)/H(+)‐Exchanger Family as Novel Prognostic Biomarkers in Colorectal Cancer,” Journal of Oncology 2021 (2021): 3241351. 34759967 10.1155/2021/3241351 PMC8575632

[27] B. Chen , M. S. Khodadoust , C. L. Liu , A. M. Newman , and A. A. Alizadeh , “Profiling Tumor Infiltrating Immune Cells With CIBERSORT,” Methods in Molecular Biology 1711 (2018): 243–259. 29344893 10.1007/978-1-4939-7493-1_12 PMC5895181

[28] M. D. Wilkerson and D. N. Hayes , “ConsensusClusterPlus: A Class Discovery Tool With Confidence Assessments and Item Tracking,” Bioinformatics 26 (2010): 1572–1573. 20427518 10.1093/bioinformatics/btq170 PMC2881355

[29] M. E. Ritchie , B. Phipson , D. Wu , et al., “Limma Powers Differential Expression Analyses for RNA‐Sequencing and Microarray Studies,” Nucleic Acids Research 43 (2015): e47. 25605792 10.1093/nar/gkv007 PMC4402510

[30] C. Zhang , Z. Zhang , Z. Zhang , et al., “The Landscape of m(6)A Regulators in Small Cell Lung Cancer: Molecular Characteristics, Immuno‐Oncology Features, and Clinical Relevance,” Molecular Cancer 20 (2021): 122. 34579719 10.1186/s12943-021-01408-5 PMC8474928

[31] B. Ru , C. N. Wong , Y. Tong , et al., “TISIDB: An Integrated Repository Portal for Tumor‐Immune System Interactions,” Bioinformatics 35 (2019): 4200–4202. 30903160 10.1093/bioinformatics/btz210

[32] Gene Ontology Consortium , “The Gene Ontology Resource: 20 Years and Still GOing Strong,” Nucleic Acids Research 47 (2019): D330–D338. 30395331 10.1093/nar/gky1055 PMC6323945

[33] M. Kanehisa , M. Furumichi , M. Tanabe , Y. Sato , and K. Morishima , “KEGG: New Perspectives on Genomes, Pathways, Diseases and Drugs,” Nucleic Acids Research 45 (2017): D353–D361. 27899662 10.1093/nar/gkw1092 PMC5210567

[34] Y. Jin , Z. Wang , D. He , Y. Zhu , X. Chen , and K. Cao , “Identification of Novel Subtypes Based on ssGSEA in Immune‐Related Prognostic Signature for Tongue Squamous Cell Carcinoma,” Cancer Medicine 10 (2021): 8693–8707. 34668665 10.1002/cam4.4341 PMC8633230

[35] A. Liberzon , C. Birger , H. Thorvaldsdóttir , M. Ghandi , J. P. Mesirov , and P. Tamayo , “The Molecular Signatures Database (MSigDB) Hallmark Gene Set Collection,” Cell Systems 1 (2015): 417–425. 26771021 10.1016/j.cels.2015.12.004 PMC4707969

[36] R. Tibshirani , “The Lasso Method for Variable Selection in the Cox Model,” Statistics in Medicine 16 (1997): 385–395. 9044528 10.1002/(sici)1097-0258(19970228)16:4<385::aid-sim380>3.0.co;2-3

[37] X. Robin , N. Turck , A. Hainard , et al., “pROC: An Open‐Source Package for R and S+ to Analyze and Compare ROC Curves,” BMC Bioinformatics 12 (2011): 77. 21414208 10.1186/1471-2105-12-77 PMC3068975

[38] W. J. Pereira , F. M. Almeida , D. Conde , et al., “Asc‐Seurat: Analytical Single‐Cell Seurat‐Based Web Application,” BMC Bioinformatics 22 (2021): 556. 34794383 10.1186/s12859-021-04472-2 PMC8600690

[39] D. Aran , A. P. Looney , L. Liu , et al., “Reference‐Based Analysis of Lung Single‐Cell Sequencing Reveals a Transitional Profibrotic Macrophage,” Nature Immunology 20 (2019): 163–172. 30643263 10.1038/s41590-018-0276-y PMC6340744

[40] B. J. Schneider , J. Naidoo , B. D. Santomasso , et al., “Management of Immune‐Related Adverse Events in Patients Treated With Immune Checkpoint Inhibitor Therapy: ASCO Guideline Update,” Journal of Clinical Oncology 39 (2021): 4073–4126. 34724392 10.1200/JCO.21.01440

[41] G. Yu , L. G. Wang , Y. Han , and Q. Y. He , “clusterProfiler: An R Package for Comparing Biological Themes Among Gene Clusters,” Omics 16 (2012): 284–287. 22455463 10.1089/omi.2011.0118 PMC3339379

[42] D. Szklarczyk , A. L. Gable , K. C. Nastou , et al., “The STRING Database in 2021: Customizable Protein‐Protein Networks, and Functional Characterization of User‐Uploaded Gene/Measurement Sets,” Nucleic Acids Research 49 (2021): D605–D612. 33237311 10.1093/nar/gkaa1074 PMC7779004

[43] Q. Cheng , X. Chen , H. Wu , and Y. Du , “Three Hematologic/Immune System‐Specific Expressed Genes Are Considered as the Potential Biomarkers for the Diagnosis of Early Rheumatoid Arthritis Through Bioinformatics Analysis,” Journal of Translational Medicine 19 (2021): 18. 33407587 10.1186/s12967-020-02689-y PMC7789535

[44] X. Li , W. Li , X. Xie , et al., “ROS Regulate Rotenone‐Induced SH‐SY5Y Dopamine Neuron Death Through Ferroptosis‐Mediated Autophagy and Apoptosis,” Molecular Neurobiology 62 (2025): 9271–9289. 40097764 10.1007/s12035-025-04824-6

[45] X. Han , B. Han , Y. Zhao , et al., “Rosmarinic Acid Attenuates Rotenone‐Induced Neurotoxicity in SH‐SY5Y Parkinson's Disease Cell Model Through Abl Inhibition,” Nutrients 14 (2022): 3508. 36079767 10.3390/nu14173508 PMC9460683

[46] S. Lotankar , K. S. Prabhavalkar , and L. K. Bhatt , “Biomarkers for Parkinson's Disease: Recent Advancement,” Neuroscience Bulletin 33 (2017): 585–597. 28936761 10.1007/s12264-017-0183-5 PMC5636742

[47] L. Mahoney‐Sánchez , H. Bouchaoui , S. Ayton , D. Devos , J. A. Duce , and J. C. Devedjian , “Ferroptosis and Its Potential Role in the Physiopathology of Parkinson's Disease,” Progress in Neurobiology 196 (2021): 101890. 32726602 10.1016/j.pneurobio.2020.101890

[48] B. R. Stockwell , J. P. Friedmann Angeli , H. Bayir , et al., “Ferroptosis: A Regulated Cell Death Nexus Linking Metabolism, Redox Biology, and Disease,” Cell 171 (2017): 273–285. 28985560 10.1016/j.cell.2017.09.021 PMC5685180

[49] Z. Lin , J. Song , Y. Gao , et al., “Hypoxia‐Induced HIF‐1α/lncRNA‐PMAN Inhibits Ferroptosis by Promoting the Cytoplasmic Translocation of ELAVL1 in Peritoneal Dissemination From Gastric Cancer,” Redox Biology 52 (2022): 102312. 35447413 10.1016/j.redox.2022.102312 PMC9043498

[50] X. Jiang , B. R. Stockwell , and M. Conrad , “Ferroptosis: Mechanisms, Biology and Role in Disease,” Nature Reviews. Molecular Cell Biology 22 (2021): 266–282. 33495651 10.1038/s41580-020-00324-8 PMC8142022

[51] F. Ursini and M. Maiorino , “Lipid Peroxidation and Ferroptosis: The Role of GSH and GPx4,” Free Radical Biology & Medicine 152 (2020): 175–185. 32165281 10.1016/j.freeradbiomed.2020.02.027

[52] H. Du , B. Chen , N. L. Jiao , Y. H. Liu , S. Y. Sun , and Y. W. Zhang , “Elevated Glutathione Peroxidase 2 Expression Promotes Cisplatin Resistance in Lung Adenocarcinoma,” Oxidative Medicine and Cellular Longevity 2020 (2020): 7370157. 32215178 10.1155/2020/7370157 PMC7079220

[53] K. Bedard and K. H. Krause , “The NOX Family of ROS‐Generating NADPH Oxidases: Physiology and Pathophysiology,” Physiological Reviews 87 (2007): 245–313. 17237347 10.1152/physrev.00044.2005

[54] K. M. Scott , “B Lymphocytes in Parkinson's Disease,” Journal of Parkinson's Disease 12 (2022): S75–S81. 10.3233/JPD-223418 PMC9535585 35938259

[55] C. Wakade , B. Giri , A. Malik , et al., “Niacin Modulates Macrophage Polarization in Parkinson's Disease,” Journal of Neuroimmunology 320 (2018): 76–79. 29759143 10.1016/j.jneuroim.2018.05.002

[56] T. Duan , Y. Du , C. Xing , H. Y. Wang , and R. F. Wang , “Toll‐Like Receptor Signaling and Its Role in Cell‐Mediated Immunity,” Frontiers in Immunology 13 (2022): 812774. 35309296 10.3389/fimmu.2022.812774 PMC8927970

[57] W. Gao , X. Wang , Y. Zhou , X. Wang , and Y. Yu , “Autophagy, Ferroptosis, Pyroptosis, and Necroptosis in Tumor Immunotherapy,” Signal Transduction and Targeted Therapy 7 (2022): 196. 35725836 10.1038/s41392-022-01046-3 PMC9208265

[58] N. Xing , Z. Dong , Q. Wu , et al., “Identification of Ferroptosis Related Biomarkers and Immune Infiltration in Parkinson's Disease by Integrated Bioinformatic Analysis,” BMC Medical Genomics 16 (2023): 55. 36918862 10.1186/s12920-023-01481-3 PMC10012699

[59] J. Joo , J. Jeong , and H. J. Park , “Blood Biomarkers in Patients With Parkinson's Disease: A Review in Context of Anesthetic Care,” Diagnostics (Basel) 13 (2023): 693. 36832181 10.3390/diagnostics13040693 PMC9955162

[60] L. Huang , X. Sun , Q. Zuo , et al., “A pH‐Responsive PROTAC‐Based Nanosystem Triggers Tumor‐Specific Ferroptosis to Construct In Situ Tumor Vaccines,” Materials Today Bio 31 (2025): 101523. 10.1016/j.mtbio.2025.101523 PMC11810845 39935894

[61] C. Guillerey , “NK Cells in the Tumor Microenvironment,” Advances in Experimental Medicine and Biology 1273 (2020): 69–90. 33119876 10.1007/978-3-030-49270-0_4

[62] T. Zeng , Y. Zhou , Y. Yu , et al., “rmMANF Prevents Sepsis‐Associated Lung Injury via Inhibiting Endoplasmic Reticulum Stress‐Induced Ferroptosis in Mice,” International Immunopharmacology 114 (2023): 109608. 36700778 10.1016/j.intimp.2022.109608

[63] G. P. Williams , A. M. Schonhoff , A. Jurkuvenaite , N. J. Gallups , D. G. Standaert , and A. S. Harms , “CD4 T Cells Mediate Brain Inflammation and Neurodegeneration in a Mouse Model of Parkinson's Disease,” Brain 144 (2021): 2047–2059. 33704423 10.1093/brain/awab103 PMC8370411

[64] V. Brochard , B. Combadière , A. Prigent , et al., “Infiltration of CD4+ Lymphocytes Into the Brain Contributes to Neurodegeneration in a Mouse Model of Parkinson Disease,” Journal of Clinical Investigation 119 (2009): 182–192. 19104149 10.1172/JCI36470 PMC2613467

[65] J. Liu , Y. Liu , Q. Zhang , et al., “Rotenone Induces Ferroptosis and Neurotoxicity Through Inhibition of SIRT1‐Nrf2‐Ferroportin 1/GPX4 Pathways in SH‐SY5Y Cells and Mice,” Chemico‐Biological Interactions 421 (2025): 111763. 41038426 10.1016/j.cbi.2025.111763

[66] T. Wang , C. Li , B. Han , et al., “Neuroprotective Effects of Danshensu on Rotenone‐Induced Parkinson's Disease Models In Vitro and In Vivo,” BMC Complementary Medicine and Therapies 20 (2020): 20. 32020857 10.1186/s12906-019-2738-7 PMC7076814

[67] Z. Huang , L. Zhou , B. Liu , X. Li , and Y. Sang , “Endoplasmic Reticulum Stress Aggravates Ferroptosis via PERK/ATF4/HSPA5 Pathway in UUO‐Induced Renal Fibrosis,” Frontiers in Pharmacology 16 (2025): 1545972. 40255561 10.3389/fphar.2025.1545972 PMC12006179

[68] C. Philippe , M. Jaud , K. Féral , et al., “Pivotal Role of the Endoplasmic Reticulum Stress‐Related XBP1s/miR‐22/SIRT1 Axis in Acute Myeloid Leukemia Apoptosis and Response to Chemotherapy,” Leukemia 38 (2024): 1764–1776. 38909090 10.1038/s41375-024-02321-8 PMC11286524

[69] H. Lu , B. Zhang , T. Yin , et al., “Ferroptosis‐Related Immune Genes in Hematological Diagnosis of Parkinson's Diseases,” Molecular Neurobiology 60 (2023): 6395–6409. 37452932 10.1007/s12035-023-03468-8

[70] S. K. Ryan , M. Zelic , Y. Han , et al., “Microglia Ferroptosis Is Regulated by SEC24B and Contributes to Neurodegeneration,” Nature Neuroscience 26 (2023): 12–26. 36536241 10.1038/s41593-022-01221-3 PMC9829540

