# Supplementary material for: Ferroptosis‐Based Peripheral Immune Dysregulation and Diagnostic Signatures in Parkinson's Disease: An RNA Transcriptomic and Single‐Cell Immune Sequencing Analysis
Source: FASEB J. 2026 Aug 2;40(15):e72144. doi: 10.1096/fj.202503867R (PMC13430149; doi:10.1096/fj.202503867R)
Supplement: Supplementary file 1 — Figure S1: Chromosome location of the genes. [file FSB2-40-e72144-s005.docx]

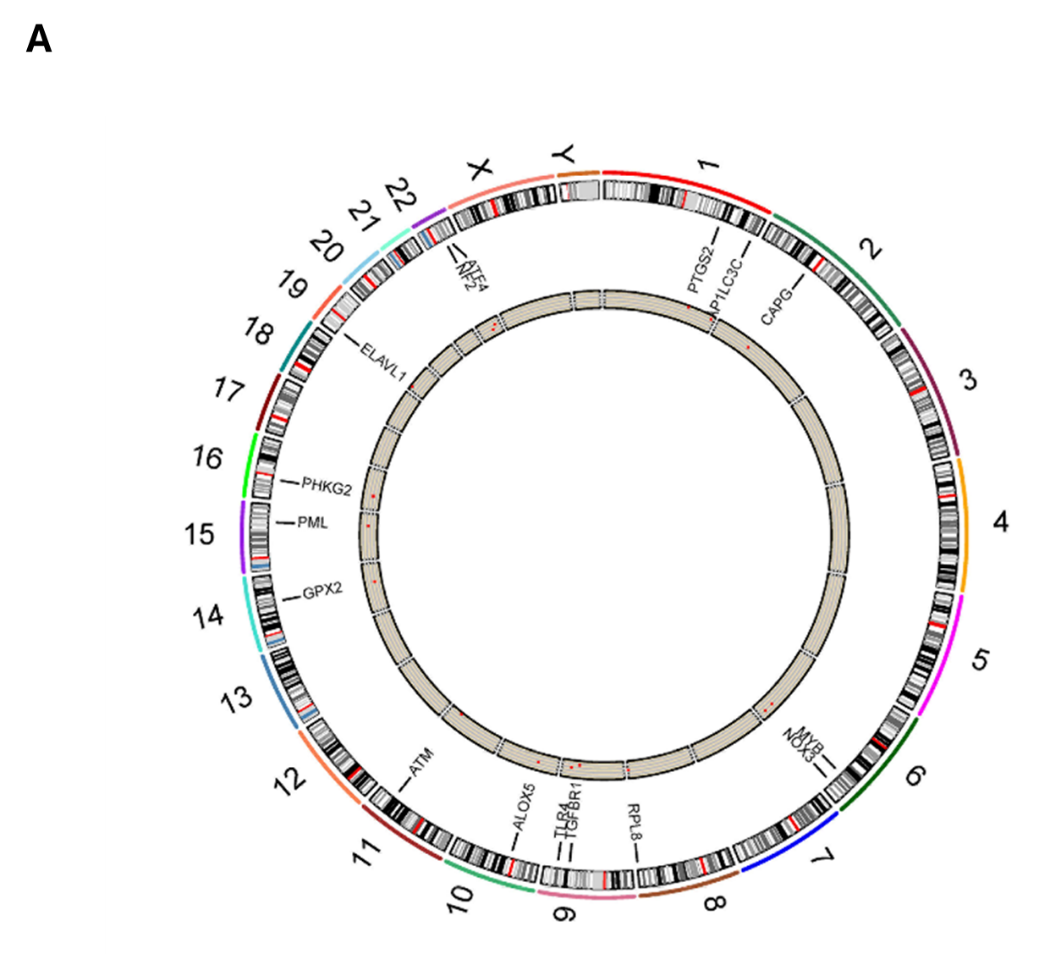
**Figure S1** Chromosome location of the genes.


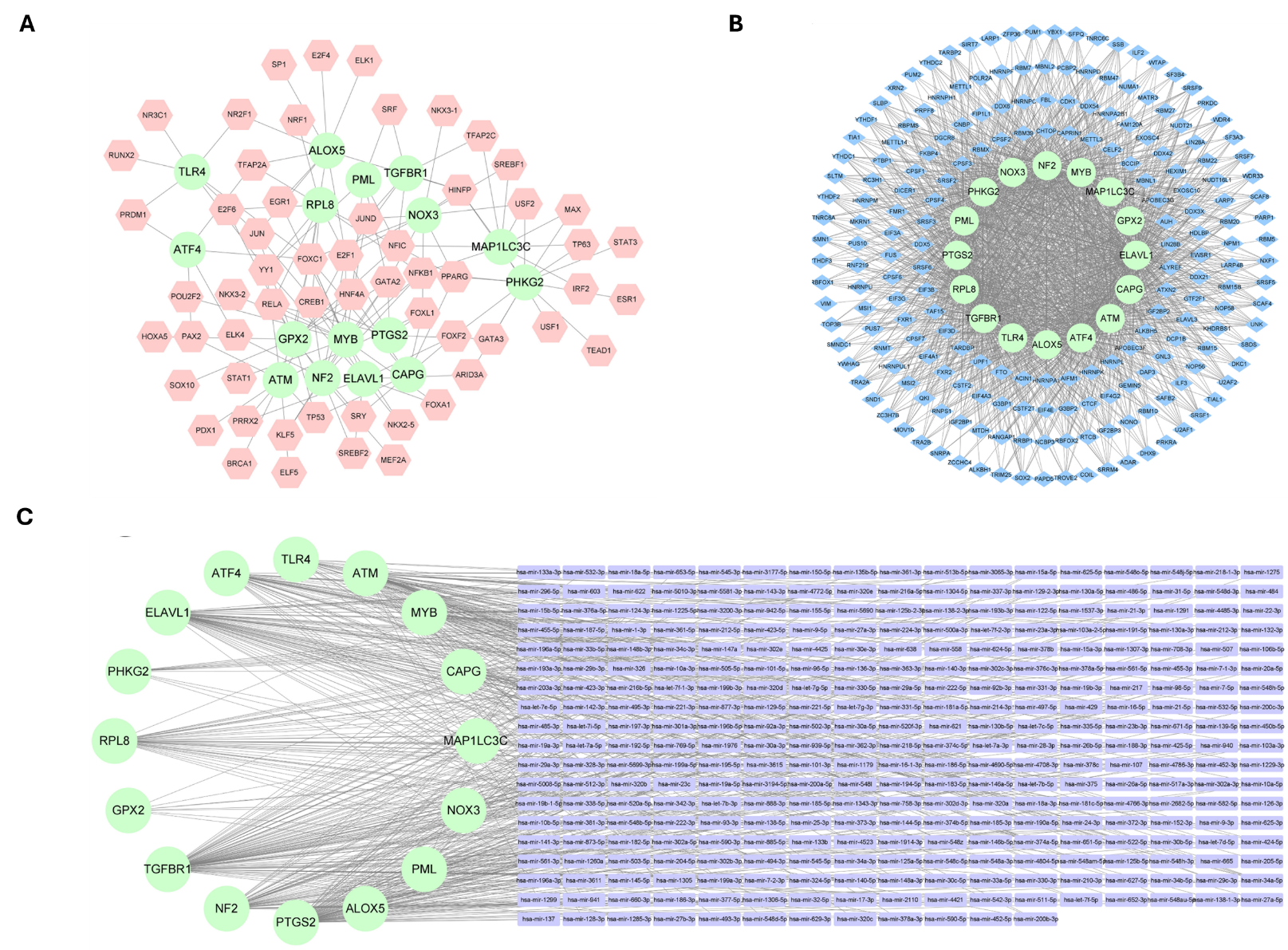
**Figure S2 The interaction network of AEFRGs based on data set GSE6613.** (A). AEFRG-TF interaction network; (B). AEFRG- RBP interaction network; (C). AEFRG-miRNA interaction network. The green circle represents AEFRGs. TF, transcription factor; RBP, RNA binding protein.
